# New software for automated cilia detection in cells (ACDC)

**DOI:** 10.1186/s13630-019-0061-z

**Published:** 2019-08-01

**Authors:** Max C. Lauring, Tianqi Zhu, Wei Luo, Wenqi Wu, Feng Yu, Derek Toomre

**Affiliations:** 1 Department of Cell Biology, Yale University School of Medicine, New Haven, CT 06510 USA; 2 College of Biomedical Engineering and Instrument Science, Zhejiang University, Hangzhou, 310027 Zhejiang China

**Keywords:** Primary cilia, Image analysis, Software, Automated cilia analysis, False positive, False negative, F1 score

## Abstract

**Background:**

Primary cilia frequency and length are key metrics in studies of ciliogenesis and ciliopathies. Typically, quantitative cilia analysis is done manually, which is very time-consuming. While some open-source and commercial image analysis software applications can segment input data, they still require the user to optimize many parameters, suffer from user bias, and often lack rigorous performance quality assessment (e.g., false positives and false negatives). Further, optimal parameter combinations vary in detection accuracy depending on cilia reporter, cell type, and imaging modality. A good automated solution would analyze images quickly, robustly, and adaptably—across different experimental data sets—without significantly compromising the accuracy of manual analysis.

**Methods:**

To solve this problem, we developed a new software for automated cilia detection in cells (ACDC). The software operates through four main steps: image importation, pre-processing, detection auto-optimization, and analysis. From a data set, a representative image with manually selected cilia (i.e., Ground Truth) is used for detection auto-optimization based on four parameters: signal-to-noise ratio, length, directional score, and intensity standard deviation. Millions of parameter combinations are automatically evaluated and optimized according to an accuracy ‘F1’ score, based on the amount of false positives and false negatives. Afterwards, the optimized parameter combination is used for automated detection and analysis of the entire data set.

**Results:**

The ACDC software accurately and adaptably detected nuclei and primary cilia across different cell types (NIH3T3, RPE1), cilia reporters (AcTub, Smo-GFP, Arl13b), and image magnifications (60×, 40×). We found that false-positive and false-negative rates for Arl13b-stained cilia were 1–6%, yielding high F1 scores of 0.96–0.97 (max. = 1.00). The software detected significant differences in mean cilia length between control and cytochalasin D-treated cell populations and could monitor dynamic changes in cilia length from movie recordings. Automated analysis offered up to a 96-fold speed enhancement compared to manual analysis, requiring around 5 s/image, or nearly 18,000 cilia analyzed/hour.

**Conclusion:**

The ACDC software is a solution for robust automated analysis of microscopic images of ciliated cells. The software is extremely adaptable, accurate, and offers immense time-savings compared to traditional manual analysis.

**Electronic supplementary material:**

The online version of this article (10.1186/s13630-019-0061-z) contains supplementary material, which is available to authorized users.

## Background

Primary cilia are highly conserved, rod-like sensory organelles that protrude from the surface of nearly all mammalian cells [1]. These tiny organelles help coordinate signaling pathways during development and tissue homeostasis. Defects associated with the assembly, structure, or function of the primary cilium lead to a wide spectrum of genetic disorders collectively known as ciliopathies [2]. Additionally, abnormally low frequency or complete loss of primary cilia is commonly observed in various tumor types, such as astrocytoma/glioblastoma [3], breast cancer [4, 5], and pancreatic ductal adenocarcinoma [6]. Aberrant changes in cilia length are associated with Niemann–Pick C1 disease [7], enhanced cellular mechanosensitivity [8], and kinase inhibitor resistance in cancer cells [9]. Researchers have investigated the therapeutic potential of inducing ciliogenesis in different human cancer cell lines with drugs such as Clofibrate, Gefitinib, and Dexamethasone [10], and with drugs to increase cilia length such as cytochalasin D, an actin depolymerizing agent [11, 12]. Thus, cilia frequency (% of ciliated cells in a population) and cilia length are important quantitative metrics for studying the relationship between cilia and disease.

A common method for quantifying primary cilia frequency and length in fixed 2D and 3D cell cultures and tissue sections is immunofluorescence microscopy using antibodies against acetylated *α*-tubulin (AcTub) or Arl13b [13–16]. For in vitro cell culture studies, specimens are prepared so that primary cilia are lying flat along the coverslip. Acquiring these types of data requires analysis of microscopy images of ciliated cells. Typically, after image acquisition, images are manually analyzed using simple line measurement tools associated with the microscope software or a generic image analysis program such as ImageJ (http://rsbweb.nih.gov/ij/). Because this process can be time-consuming and susceptible to user bias, there has been a push for the development of practical, automated image analysis software over the past two decades. Automated image analysis algorithms can help biologists quantify information in an efficient, objective, and reproducible manner. Accurate automated performance can even detect small changes that are too subtle, or too tedious, for the human visual system to assess [17].

While existing modular open-source and commercial image analysis solutions allow for the implementation of complex image analysis pipelines [18], they leave the parameter adjustment of the component algorithm up to the user. Examples of commercial software include MetaMorph, Amira (Visage Imaging), Volocity (PerkinElmer), Imaris (Bitplane Scientific Software), NIS-Elements, SlideBook, ImagePro Plus (Media Cybernetics), and ZEN (Zeiss). Examples of open-source image analysis software include BioImageXD [19], Icy [20], Fiji [21], Vaa3D [22], CellProfiler [23], 3D Slicer [24], Reconstruct [25], FluoRender [26], OsiriX [27], IMOD [28], and ImageJ [29]. Numerous plug-ins and macros have been written for ImageJ/Fiji for measuring features such as nuclear segmentation, cilia intensity, total volume, average intensity, and cilia length [10, 30–33]. However, the user must adjust multitudes of parameters to find an optimal parameter combination—a cumbersome and bias-prone process—that is likely specific to a certain cell type, treatment condition, cilia reporter, or imaging setting.

Furthermore, while ‘plug-ins’ that provide bespoke solutions have been made available, little to no corresponding validation data or rigorous performance analysis has been made available in the context of primary cilia analysis. Namely, there is virtually no validation to show the percent of false positives or false negatives, and thus the accuracy of such bespoke solutions is unknown. Researchers have used programs such as IN Cell Analyzer 2000 Imaging system and CytoShop HCS analysis software (Beckman Coulter) to automatically analyze images of ciliated cells [10, 11], but these studies do not appear to (i) analyze images automatically based on training from a prior Ground Truth or (ii) easily adapt optimization parameters towards different experimental conditions.

Here we developed ‘ACDC’ (Automated Cilia Detection in Cells), a user-friendly, MATLAB-based solution specifically for the detection and analysis of primary cilia. The ACDC software accurately detects and measures nuclei and primary cilia in 2D microscopy images and then exports these data to Microsoft Excel files. For cilia detection, the software utilizes only four parameters, which address nuances in cilia intensity, shape, and length. The software provides an auto-optimization feature that uses a single, manually marked-up representative image for training; this is a very quick initial procedure. Subsequently, an optimal parameter combination (from over 5.5 million possible combinations) is chosen for the automated analysis of the representative image itself and every other image in the data set. Because of this large parameter space and auto-optimization process, ACDC can be used to analyze vastly different data sets with differing experimental conditions. We demonstrate this capability using two different cell lines, three different cilia reporters, and two different image magnifications. We also report the false-positive and false-negative rates for each of these conditions and then calculate their respective accuracy scores (F1 scores). Lastly, we demonstrate that ACDC can be used for accurate, wide-scale length measurement of ciliated cells, and report 28- to 96-fold increases in the number of cilia analyzed per unit time for fully automated analysis, relative to manual analysis.

## Methods

### Tissue cell culture, stable transfection, and reagents

htert-RPE1 cells (ATCC) were cultured in DMEM/F-12 (Invitrogen) with 10% FBS (Sigma-Aldrich), 2 mM sodium pyruvate (Invitrogen), 100 U/ml penicillin–streptomycin (Invitrogen), and MEM non-essential amino acids (Invitrogen). NIH3T3 cells (BPS Bioscience) were cultured in DMEM (Invitrogen) with 10% BS (Sigma-Aldrich), 2 mM sodium pyruvate (Invitrogen), 100 U/ml penicillin–streptomycin (Invitrogen), and MEM non-essential amino acids (Invitrogen). htert-RPE1-Smo-GFP stable cell lines were generated as described previously [34]. For cytochalasin D treatment, cells were grown on glass coverslips until confluency, serum-starved, and then grown in serum starvation media (0% FBS) supplemented with 100 nM cytochalasin D (Sigma-Aldrich) overnight. The following antibodies were used: acetylated *α*-tubulin (mouse monoclonal; Sigma-Aldrich) and Arl13b (mouse monoclonal; NeuroMab). The following dyes were used: goat anti-mouse IgG (H + L) secondary antibody Oregon Green 488 (Invitrogen) and Hoechst 33342, trihydrochloride, trihydrate (Invitrogen).

### Immunofluorescence

Cells were plated on 12 mm glass coverslips (Fisherbrand) in 24-well plates and, upon confluency, incubated in serum starvation media containing 0% FBS for 48 h to induce ciliogenesis. Cells were then washed with PBS (2 × 1 min), fixed with 4% paraformaldehyde in PBS for 10 min at room temperature, washed with PBS (3 × 1 min), permeabilized with 0.1% Triton X-100 for 10 min, washed with PBS with 0.3% Tween 20 (3 × 1 min), and blocked in 5% bovine serum albumin (BSA) with 0.3% Tween 20 for 30 min. Cells were then incubated for 1 h in primary antibody against Arl13b (1:300) or acetylated *α*-tubulin (1:400) in a wet chamber. Following several more washes in blocking buffer, cells were incubated in dye-conjugated secondary antibodies and Hoechst dye for 30 min, washed again, and mounted on a coverslide with ProLong gold antifade reagent (Invitrogen).

### Microscopy image acquisition and analysis

Fixed cells were imaged on a Yokogawa-type Spinning-Disk Confocal Microscope (SDCM, PerkinElmer). The SDCM is mounted on an inverted microscope base (IX-71, Olympus) equipped with a 512 × 512 pixels electron-multiplying charge-coupled device camera (Hamamatsu Photonics) and a temperature-controlled stage set (custom built). The SDCM is controlled by the Ultraview ERS software (PerkinElmer) and the cells were imaged via a 60 × 1.4 NA oil objective lens with a pixel size of 0.143 µm using 5 solid-state lasers 405-, 488-, 561-, 594-, and 640-nm (Melles Griot). Exposure times were typically between 100 and 140 ms. Z-stacks of images were taken at a separation of 0.5 µm. Images were acquired using Volocity software (PerkinElmer), which employs a maximum intensity projection (MIP) when creating 2D images from z-stacks. This process occurs prior to any image importation into the ACDC software. For live-cell imaging, RPE-Smo-GFP cells were grown in MatTek dishes for 2 days, then grown in serum starvation media with 100 nM cytochalasin D, and then imaged by SDCM. Cells were also imaged on an EVOS fluorescent microscope using the Invitrogen EVOS FL Auto 2 Cell Imaging System. A 40 × 0.95 NA air objective lens was used with a pixel size of 0.1787 µm. EVOS light cubes used in this study include DAPI (357/44 nm Excitation; 447/60 nm Emission) and GFP (470/22 nm Excitation; 510/42 nm Emission). In figures that analyzed hundreds of images from a single experiment, images were acquired in an automated manner based on nuclei autofocusing using the SDCM or EVOS microscope. Images were later analyzed manually, automatedly, and semi-automatically with the ACDC software (v0.81). The software was used on an Alienware laptop by Dell (Windows 10 Pro; Intel^®^ Core™ i7-6700HQ CPU @ 2.60 GHz; Intel^®^ HD Graphics 530 and NVIDIA GeForce GTX 1070).

### Statistical analysis

Statistical significance was calculated using two-tailed Student’s *t* test. *p < 0.05, ***p < 0.001. Figures containing cilia count and length measurements are expressed as mean ± standard deviation. Statistical tests were performed in Microsoft Excel and GraphPad Prism 7.0 software.

### ACDC software: image pre-processing for primary cilia detection

The algorithm is based on the assumption that fluorophores with different wavelengths are used to image primary cilia and cell nuclei. Potential candidates for cilia are identified in a pre-processing step using the green channel. In a subsequent step, biologically relevant measurements are extracted for each of the confirmed detected candidates. Similarly, nuclei are detected and segmented using the blue channel.

Five pre-processing steps are applied to the green channel to generate potential cilia candidates. To achieve a reliable detection of such fine and small structures in a robust fashion, it is necessary to reduce image noise and enhance the image contrast. In the first two steps, we apply contrast-limited adaptive histogram equalization [35], followed by Gaussian filtering to smooth the image and suppress background noise. In this context, a basic noise removal strategy based on linear filtering is sufficient, as we aim to remove some high-frequency signals that could generate spurious candidate detections. The size of the Gaussian filter is adjusted based on the resolution of the acquired images. In the third step, a top-hat transformation is applied to the image, serving as a grayscale morphological operation that preserves small, bright, linear structures. Because there is variation between the background intensities of each candidate, a threshold based on global intensity values cannot be directly set to extract objects separately from the background. A top-hat transformation with a disk-shaped structure element of radius 4 corrects for non-uniform background intensities and preserves bright objects against a further suppressed background. In the fourth step, pixel locations that have a high likelihood of corresponding to candidate cilia are identified by binarization using adaptive thresholding. Due to differences in brightness between candidate cilia, some low-brightness candidates may be lost if global thresholding is used to binarize the image. Adaptive thresholding helps account for this phenomenon by changing the threshold dynamically over the entire image. We apply the Gaussian filter from before as an adaptive thresholding method to binarize the image:

$$ I_{\text{bw}} = I_{\text{tophat}} > \left( {2*f\left( {I_{\text{tophat}} , \sigma = 12} \right)} \right), $$

where $I_{\text{tophat}}$ denotes the result of the top-hat transformation, and $I_{\text{bw}}$ denotes the result of the binarization using adaptive threshold. The last step of pre-processing comprises preliminary selection. In the resulting binary image, many connected components will be brighter than surrounding background pixels, and some of these components could be false positives. Preliminary selection uses a parameter called directional score (explained in the next section) to reject any connected components that do not meet a threshold of 0.01, the absolute lowest value for directional score. After pre-processing, the raw image and the final binary image are used for further detection auto-optimization using parameters based on shape, length, and intensity. The optimal value for each parameter is automatically determined.

### Parameters for auto-optimized cilia detection

The directional score parameter, which was introduced in the previous section, is used again to ensure the detection of rod-like candidates. Because the majority of actual cilia are rod-shaped with a clear direction, we use rod-shaped structural elements of varying degrees to conduct morphological erosion operations on connected components. A connected component is considered a false-positive candidate if its calculated directional score is below the set threshold:

$$ {\text{Score}}^{\left( i \right)} = \mathop {\hbox{max} }\limits_{k} \left( {\frac{{\left| {{\text{CC}}^{\left( i \right)} { \ominus }{\text{LE}}_{k} } \right|}}{{\left| {{\text{CC}}^{\left( i \right)} } \right|}}} \right)      \quad k = \left\{ {1,2, \ldots 11,12} \right\}, $$

where ${\text{CC}}^{\left( i \right)}$ is the i-th connected component of $I_{\text{bw}}$, ${ \ominus }$ is the morphological erosion operation, ${\text{LE}}_{k}$ is a rod-shaped structure element with length $L = \frac{{\rm{min} \left( {{\text{width,}}\,\,{\text{height}}} \right)}}{50}$, width 1, degree 15*k, and the function $\left| \cdot \right|$ indicates the number of pixels with a value of 1.

The minimum length parameter describes the length of a candidate cilium’s skeleton. We use a thinning algorithm [36] to generate a raw skeleton. In the instance that there are several branches from one skeleton (due to irregular binary block shape), the longest branch is chosen as the candidate’s skeleton. Lastly, a cubic spline interpolation is applied to smooth out any coarse skeletons, resulting in more accurate candidate lengths. Conveniently, this smoothing feature is also applied to manual analysis.

The signal-to-noise ratio (SNR) parameter accounts for the fact that the intensities of true cilia are usually brighter than surrounding background, even though absolute intensity values may vary greatly between candidates. SNR, rather than absolute intensity, is a better parameter to distinguish true cilia from background. The signal area of a candidate includes all pixels of a connected component. The signal level is defined as the average intensity of these pixels. The background noise area is defined as the region 1 to 5 pixels radially away from a connected component. The background noise level is the average intensity of these pixels.

The standard deviation (STD) parameter accounts for intensity variations along the cilia length. After applying thresholds for SNR and directional score, there may still be some false candidates—possibly membranous structures or linear fixation artifacts—that possess high values for SNR and directional score. The STD parameter can help reject many of these false candidates. The brightness of a cilium’s center usually differs from that of a cilium’s base or tip, which translates to a larger standard deviation along the cilia length. Conversely, non-cilia objects will have less variation of pixel brightness.

### ACDC software: image pre-processing and segmentation for nuclei detection

While many sophisticated nuclei segmentation algorithms have been proposed, we have implemented a basic strategy to detect and segment nuclei using the DAPI (blue) channel. Pre-processing for nuclei detection comprises three steps. The first two steps improve the quality of image, while the third step uses an adaptive-global combined thresholding method for binarization. In the binary image, one connected component may consist of several overlapping nuclei. In brief, to solve this problem, an algorithm based on concave joints is applied to binary images to segment these connected components.

The first step of nuclei pre-processing is median filtering. The background of the blue channel is generally much cleaner than that of the green channel, which is used for cilia detection. To ensure that the edges of the nuclei remain sharp, median filtering (size 7) is used to preserve edges while removing noise:

$$ I_{b\_f} \left( {x,y} \right) = {\text{median}}\left( {\left\{ {I_{b} \left( {x + i,y + j} \right)| - s \le i,j \le s, i,j \,{\text{is interger}}} \right\}} \right), $$

where $s$ denotes the size of the filter kernel, $I_{b}$ denotes the blue channel of the raw input image, and $I_{b\_f}$ denotes the result of median filtering on the blue channel.

The second step consists of binarization using adaptive-global combined threshold. Global thresholding alone would result in incomplete nuclei, and adaptive thresholding alone may produce artifacts in dark areas of the image. Thus, we combined adaptive thresholding with global thresholding to binarize the result from the previous step. The global threshold is computed by Otsu’s method [37], denoted by $T$ in the following equations, and a Gaussian filter is applied to $I_{b\_f}$ to obtain an adaptive local threshold map [38] denoted by $M_{0}$:

$$ M_{0} = f\left( {I_{b\_f} , \sigma = s} \right), $$

$$ s = 2*{\text{floor}}\left( {\frac{{\hbox{min} \left( {H,W} \right)}}{16}} \right) + 1, $$

where ${\text{floor}}\left( \cdot \right)$ means round down operation, and $H, W$ represents the height and width of the $I_{b\_f}$, respectively. $T$ and $M_{0}$ are combined to get the final threshold map for image binarization:

$$ M_{1} = \hbox{min} \left( {1,\hbox{max} \left( {0, 0.95*\alpha *M_{0} + 0.05*T} \right)} \right), $$

$$ I_{{b\_{\text{bw}}}} = I_{b\_f} > M_{1} , $$

where $\alpha$ represents the adjustable parameter ‘sensitivity’ and $I_{{b\_{\text{bw}}}}$ represents the result of binarization using adaptive-global combined threshold.

The third step consists of region correction. After completing the first two steps, the resulting image would still contain several connected components that are nearby actual nuclei. Connected components that are too small in size tend to be artifacts. Thus, connected components with areas smaller than a set threshold are rejected. Finally, a solution for overlapping nuclei based on high curvature points, as proposed by Fan et al. [39], is used to split and segment nuclei.

## Results

### Conceptualization and framework of ACDC software

**Fig. 1:**
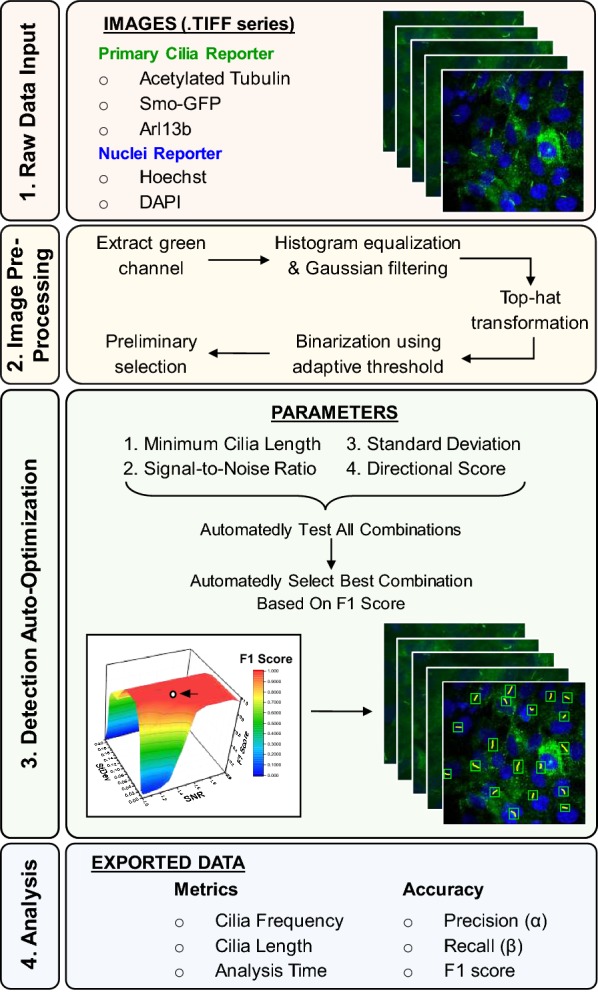
Overview flow diagram of Automated Cilia Detection in Cells (ACDC) software. The ACDC software for automatic detection of cilia comprises four main steps, which are lightly color-coded in four boxes: data input (red), pre-processing (yellow), detection (green), and analysis (blue); later figures follow this color-coding scheme. Step One: users can import hundreds of microscope images that comprise a cilia reporter and a nuclei reporter. Step Two: the images are pre-processed to facilitate formation of a binary mask. Step Three: based on a manually marked-up representative image, the software auto-optimizes the detection of true candidates by determining which combination of the four listed parameters yields the greatest accuracy score (F1 score). The parameter combination is then used to automatically detect true candidates from images of the same or similar image series. Step Four: analysis and exportation of data, which includes accuracy metrics such as false-positive (FP) and false-negative (FN) rates and typical cilia metrics such as frequency and length. The analysis time is also reported for comparison between manual, automated, and semi-automated analysis (i.e., automated analysis mode with the ability to manually correct detection errors)

**Fig. 2:**
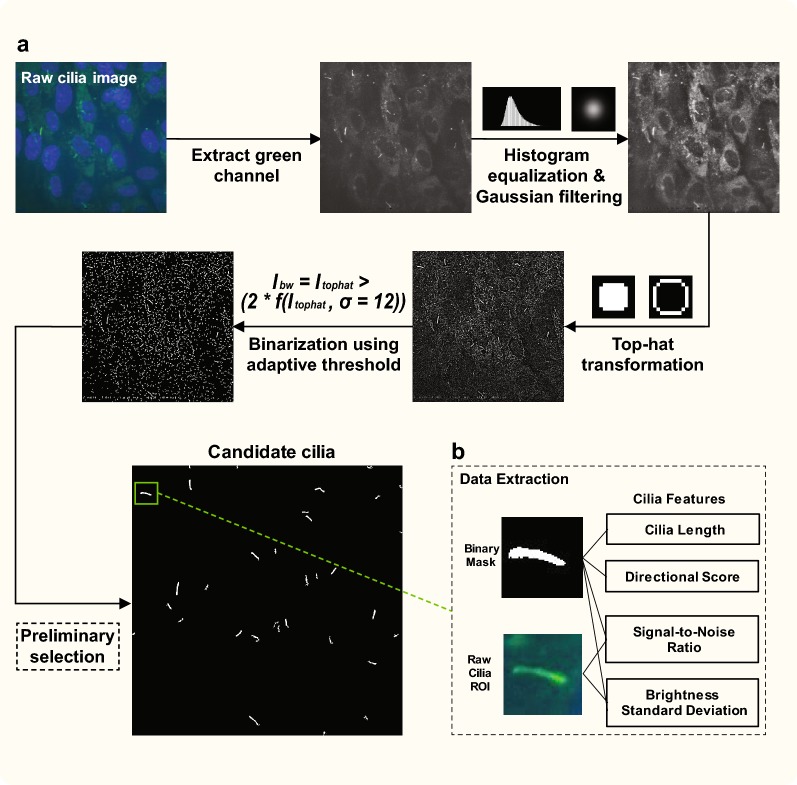
Image pre-processing and initial candidate detection. **a** Upon initiation of automated analysis, each imported raw image undergoes a series of pre-processing steps that aid in identifying prospective candidate cilia and excluding other non-relevant objects present in the image. After binarization using adaptive threshold and preliminary selection, only the most likely tens of candidate cilia will be shown. **b** The detection of true candidates can be further automatically optimized using the software’s four tuning parameters. Data pertaining to length and directional score depend only on the binary mask, while data pertaining to signal-to-noise ratio (SNR) and standard deviation (STD) depend on both the binary mask and raw cilia ROI

An overview of the resulting algorithm is presented in Fig. 1. Four primary steps are described, beginning with the importation of raw images and ending with the exportation of cilia data. The user has the option to analyze images in three modes: manual, automated, or semi-automated (i.e., corrected automated). In semi-automated mode, the user has the ability to correct for any discrepancies that arise during the automated analysis of each image. The first step of the automated analysis process involves the importation of microscope images, ideally in TIFF format. Images must have a minimum of two channels: (i) one for primary cilia such as acetylated tubulin (AcTub), Smoothened-GFP (Smo-GFP), or Arl13b, and (ii) one for nuclei such as DAPI or Hoechst stain. These reporters are just several of the many examples and are in no way exclusive. The second step of the automated analysis process focuses on the very first image in the series, which is used as a representative image for training and calibration. This begins with image pre-processing and involves five sub-steps—extraction of green channel, histogram equalization with Gaussian filtering, top-hat transformation, binarization using adaptive threshold, and preliminary candidate selection. The purpose of pre-processing, as explained in detail in the ‘Methods’ section, is to generate potential candidate cilia from the raw image and binary mask of the raw image (Fig. 2). The third step occurs after initial candidate identification and relies on automated parameter optimization to better detect true cilia. We have determined that four key parameters—(i) minimum (min.) cilia length, (ii) signal-to-noise ratio, (iii) intensity standard deviation, and (iv) directional score—are needed to distinguish actual cilia from other cellular structures. The result of the detection auto-optimization process yields the optimal combination of parameter values. In the fourth and final step, the optimal, computer-assigned parameter set is used on the representative image and the rest of the image series. Upon completion of the analysis session, cilia data can be exported to a Microsoft Excel document. Additionally, through semi-automated analysis, the user can obtain metrics about the performance of fully automated analysis such as false-positive (FP) count, false-negative (FN) count, precision (*α*), recall (*β*), F1 accuracy score {F1 = 2*αβ*/(*α* + *β*)} [40], image-specific surface plots of possible F1 scores (Additional file 1: Fig. S1), and correction analysis time. Each image requires only several seconds to automatically analyze and thus allows for efficient analysis of large data sets of ciliated cells.

### Automated detection parameters

**Fig. 3:**
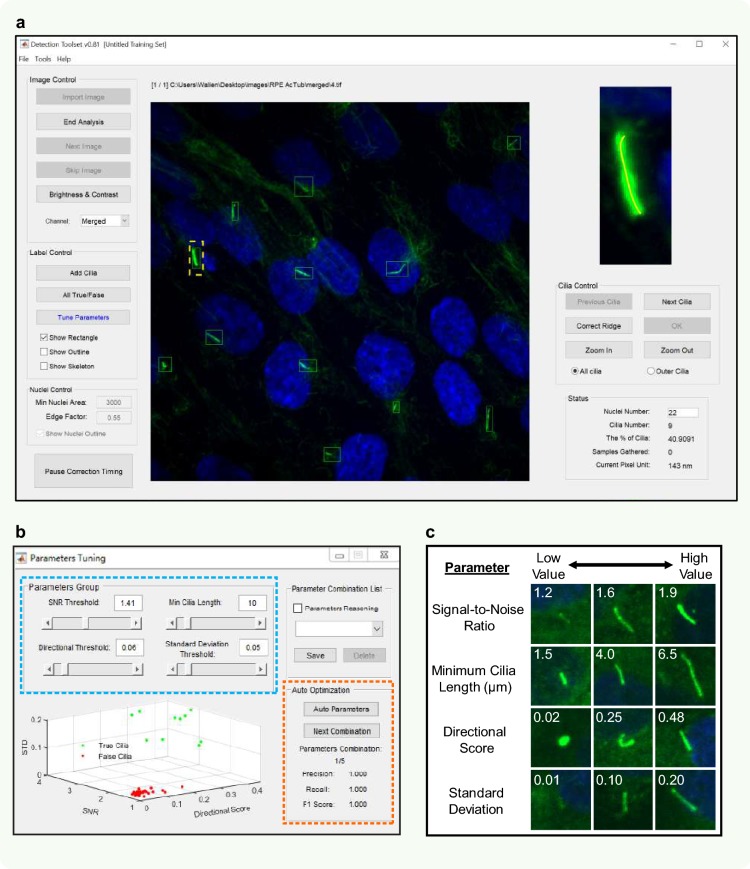
Parameters for detection optimization. **a** A screenshot of the user interface of the ACDC software. **b** The interface that appears when the “Tune Parameters” option in the left panel in part (A) is selected. The user can manipulate four parameters that aid in detection optimization of candidate cilia (blue dashed box). Signal-to-noise ratio (SNR) compares the intensity of a cilium to the intensity of surrounding background. SNR values range from 1.0 to 2.0. Minimum cilia length values range from 0 to 100 pixels. Users can assign a distance per pixel. Brightness standard deviation accounts for the distribution of different pixel intensities along the entire cilium. Standard deviation values range from 0 to 0.50. Directional score accounts for cilia curvature. Cilia with high directional scores are straighter and rod-like, while cilia with low directional scores are curvier and spherical. Directional threshold values range from 0.01 to 0.51. Higher parameter threshold values result in the rejection of more candidates. **c** Examples of primary cilia with different values for SNR, cilia length, directional threshold, and standard deviation. Typically, candidates with higher parameter values, aside from length, are more likely to be true cilia, but it is still possible for non-cilia objects such as bright aggregates to be selected

**Fig. 4:**
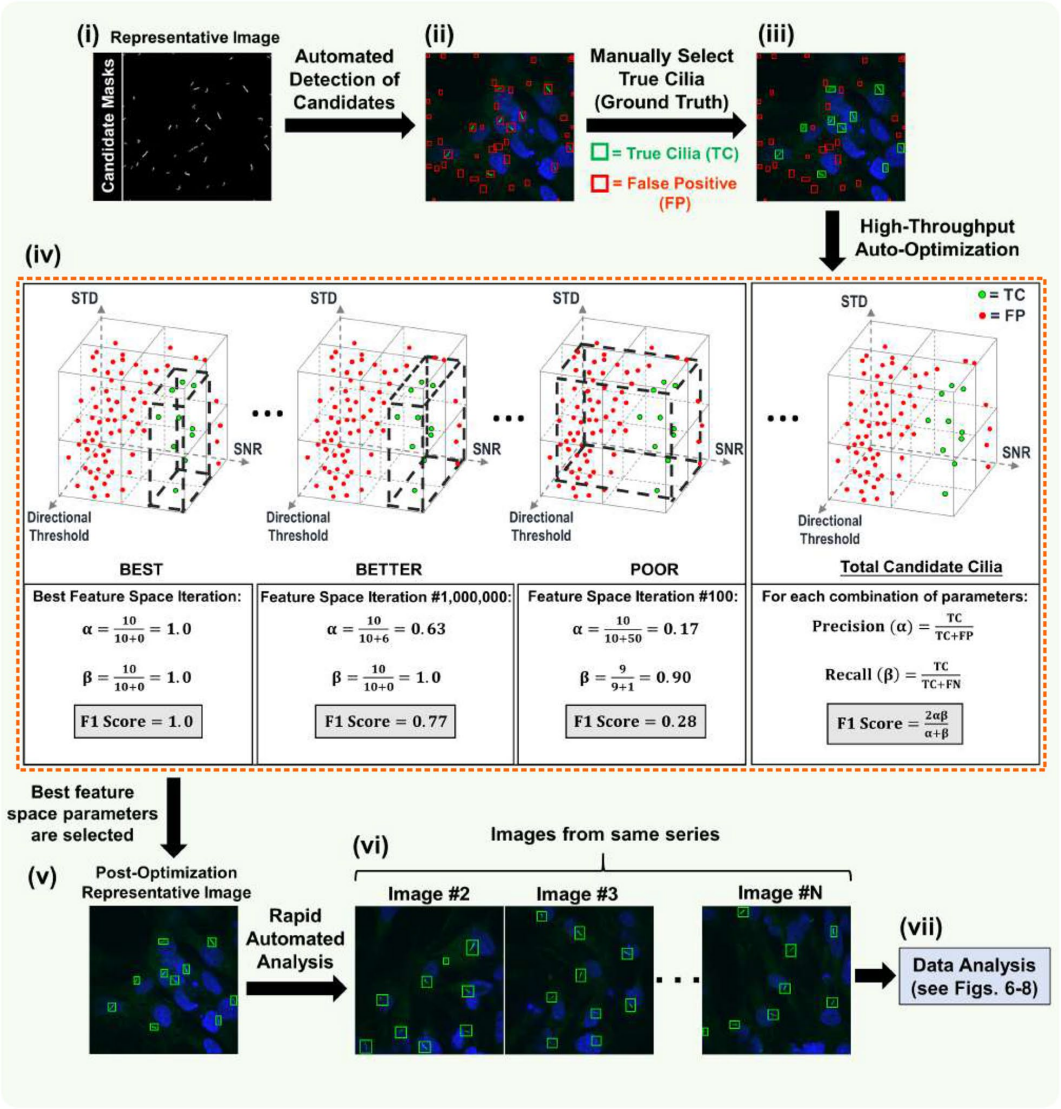
Automated detection optimization of candidate cilia. Auto-optimization of cilia detection consists of seven steps (i–vii). (**i**) The resulting image of the pre-processing step. Because this was the first image of the imported data series, it is used as a representative image for training and calibration. (**ii**) Initially, all post-binarization candidates considered to be false objects (outlined in red). (**iii**) Users must tell the software which candidates to accept by clicking on those true candidate cilia (now outlined in green). (**iv**) After the “Auto Parameters” feature is selected (see Fig. 3b; orange dashed box), the remainder of the analysis process is automated. Based on the candidates that were manually selected to be true, the software sifts through ~ 5.5 million iterations of parameter combinations and chooses the feature space (depicted as dashed black boxes) that encloses only the true candidates and excludes the other false candidates. The software determines the accuracy of each iterative trial by searching for the feature space that yields that greatest F1 score. The F1 score is a function of two variables—precision (*α*), which describes the frequency of false positives (FPs), and recall (*β*), which describes the frequency of false negatives (FNs). Here, the automatically detected true candidates are represented by ‘TC.’ (**v**–**vi**) This best feature space is used to automatically analyze the remainder of the image series. If necessary, the user has the ability to adjust the optimized feature space to their preference. (**vii**) After analysis of the last image in the series, the data are exported for further study

A screenshot of the user interface of the ACDC software is shown in Fig. 3a. Features of the “Image Control” panel include importation, analysis, adjustment of image contrast, and the ability to switch between DAPI, green, and merged channels. Detected candidates that are outlined in green boxes are considered true cilia (TC), as shown in the center display. Features of the “Label Control” panel include the ability to convert all detected true objects into detected false objects and vice versa, the ability to add a candidate to the analysis in the event of a missed detection, and the ability to visualize candidate skeletons. The interface that appears when the user selects the “Tune Parameters” option is shown in Fig. 3b. Here, the user can adjust the threshold values of the four parameters for detection optimization. Signal-to-noise ratio (SNR) compares the intensity of the cilium to the intensity of the surrounding background, as true cilia generally have high intensity values that can easily be distinguished from background intensity. SNR values range from 1.0 to 2.0. Minimum cilia length sets the threshold for long and short cilia, regardless of whether cilia are straight or curvilinear. Minimum cilia length values range from 1 pixel to 50 pixels; detected objects with lengths less than the set threshold will be rejected from detection. Directional score accounts for the curvature of cilia. Cilia with higher directional scores are straighter and more rod-like, while cilia with lower directional scores are curvier and more spherical. Directional score values range from 0.01 to 0.51. This parameter is useful in differentiating between actual cilia and other bright objects such as ciliary extracellular vesicles and endosomes that can be 0.5–2.0 μm in diameter, as the majority of primary cilia tend to be more rod-like in shape. Intensity standard deviation (STD) accounts for the distribution of the different pixel intensities along the entire cilium. Pixels that belong to true cilia should have larger standard deviations, since, in theory, these pixels are generated from a 2D-point spread function (like the Gaussian distribution) with a relatively high standard deviation. Thus, candidates with high STD values are likely to be true cilia. STD values range from 0 to 0.50, and higher STD threshold values will reject more candidates. Examples of primary cilia with low and high values for SNR, cilia length, directional score, and standard deviation are shown in Fig. 3c. Of note, the “Tune Parameters” interface in Fig. 3b offers an “Auto Parameters” feature that allows for detection auto-optimization of post-binarization candidates. The “Auto-Optimization” panel in Fig. 3b provides output metrics including precision (*α*), recall (*β*), and accuracy (F1 score), described in detail in Fig. 4.

### Detection auto-optimization based on a representative ‘Ground Truth’

The process for automated detection optimization of candidates is described in Fig. 4. Whether the user wants to analyze a single image or a series of images, the software will use the first image as the representative image to auto-calibrate parameter settings; users can save these parameter settings for future analyses of images from similar experiments. Upon importation of images, all post-binarization candidates will appear in red outlines, indicating that they are false objects that should be rejected and excluded from any analysis. These candidates comprise both true cilia and possibly non-cilia objects such as membranous structures, mitotic structures, vesicles, and fixation artifacts. Initially, the user must manually select the candidates that they consider true cilia. As a time-savings, the user does not have to manually trace candidates and only has to click a box that already automatically outlines candidates. Upon selection, these candidates will appear in green outlines, indicating that they are true cilia that should be accepted and included in the analysis. These green-outlined candidates comprise a reference standard known as the ‘Ground Truth.’ The user then selects the “Auto Parameters” feature under the Auto-Optimization panel (Fig. 3b), and from this point forward, the analysis process is completely automated. Based on the Ground Truth, the software will iteratively sift through over 5.5 million combinations of the four detection parameters until a combination is found that best selects for the candidates in the Ground Truth. The software will determine the accuracy of each iterative trial according to each trial’s F1 score, which can range from 0 (least accurate) to 1.00 (most accurate). The F1 score is a mathematical function of two variables: precision (*α*), which describes the frequency of incorporated false positives (FPs), and recall (*β*), which describes the frequency of false negatives (FNs; true cilia that were missed and overlooked for detection). In statistical analysis of binary classification, *α* is the number of correct positive results divided by the number of all positive results returned by the program, while *β* is the number of correct positive results divided by the number of all relevant samples (all samples that should have been identified as positive). Values for *α* and *β* both range from 0 (entirely FPs and FNs, respectively) to 1.00 (no FPs and FNs, respectively). Higher *α* values indicate fewer incorporated FPs, while higher *β* values indicate fewer FNs.

$$ \alpha = \frac{\text{TC}}{{{\text{TC}} + {\text{FP}}}}          \quad \beta = \frac{\text{TC}}{{{\text{TC}} + {\text{FN}}}}          \quad F1 = \frac{2\alpha \beta }{\alpha + \beta } $$

In the equations above, ‘TC’ represents only the number of actual true cilia that were detected by automated analysis, ‘FP’ represents the number of false positives, and ‘FN’ represents the number of false negatives. If there are multiple parameter combinations that yield the same maximal F1 score (see Additional file 1: Figure S1 for surface plot visualizations of this phenomenon), the software’s default is to choose the parameter combination that minimizes all parameter values while retaining that maximal F1 score. Once the best feature space (best multivariate set of parameters) is established, it is used for continuous automated analysis of the entire data set. However, the user can adjust the optimized feature space parameter thresholds to their preference at any time during the analysis session by pausing the analysis of the next image, re-opening the “Tune Parameters” interface, altering the threshold values, observing how the detection of cilia in the current image changes, and continuing the analysis of the rest of the data set (Fig. 3b). Additionally, before continuing the analysis of the next image, the user can add cilium manually to account for FNs or click the outlines of any FPs to mark them as not true cilia (i.e., they will then be in red outlines). Naturally, the trade-off here is a longer analysis time. Upon automated analysis of the last image in the series, the cilia data are exported for further study.

In the automated analysis mode of the ACDC software, the established optimized feature space is ‘locked’ and applied to every image in the series. The software usually identifies most true cilia, but the presence of FPs and FNs is possible during automated analysis, which can skew observations and conclusions. Because the software uses a Ground Truth data set for optimization, it is important that the established Ground Truth attempts to accurately reflect the entire data set population. For instance, if an image with very bright, straight cilia is used for auto-optimization, but the next image in the series has one or two low intensity, curvilinear cilia, these cilia might not be detected if they do not meet the optimized parameter thresholds, resulting in one or two FNs. There are two routes the user can pursue from here. First, the user can accept the presence of some FPs and FNs if the FP and FN rates are low enough to meet expectations. Second, during automated analysis, the user can manually correct for any FPs and FNs that they notice (i.e., semi-automated analysis). Correcting images for FPs and FNs by semi-automated analysis will yield the same detection and analysis results as manual analysis, but the trade-off is a longer analysis time than fully automated analysis. The extent to which semi-automated analysis is slower than fully automated analysis is directly correlated with the amount of FPs and FNs that need to be corrected. To correct for FPs, the user can simply click on the green outline of the detected candidate to change it to a red outline, telling the software to reject the candidate from analysis. To correct for FNs, the software has an ‘Add Cilia’ feature (Fig. 3a) that lets the user draw a box around a candidate, whereupon the software will automatically draw a yellow spline overlay that measures the candidate’s length. The yellow spline overlay of any candidate can be edited and re-drawn by the user. During manual analysis with the software, the user must draw a box around and trace over candidates that they consider to be true cilia.

It is important to note that it is possible for the auto-optimization process to yield an optimized feature space in which the F1 score < 1.00. The ideal result of the auto-optimization process, as illustrated in Fig. 4, is to end up with only the Ground Truth candidates in green outlines and every other post-binarization non-cilia candidate without an outline (i.e., rejected and excluded from detection). The alternate outcome, however, is that all the Ground Truth candidates appear in green outlines, along with several false objects with green outlines that should have been excluded. These false objects cannot be filtered out because they are too similar in intensity and shape to the Ground Truth cilia. One could manually adjust the parameter thresholds to exclude these false objects, but it would be impossible to do so without also excluding one or several Ground Truth candidates. In this instance, the user has three options: (1) simply accept any false object as part of the initial Ground Truth, (2) manually adjust the detection parameter thresholds to filter out the false objects along with one or several Ground Truth cilia, or (3) attempt to auto-optimize by choosing another representative image from the data set.

### Software performance on real samples

**Fig. 5:**
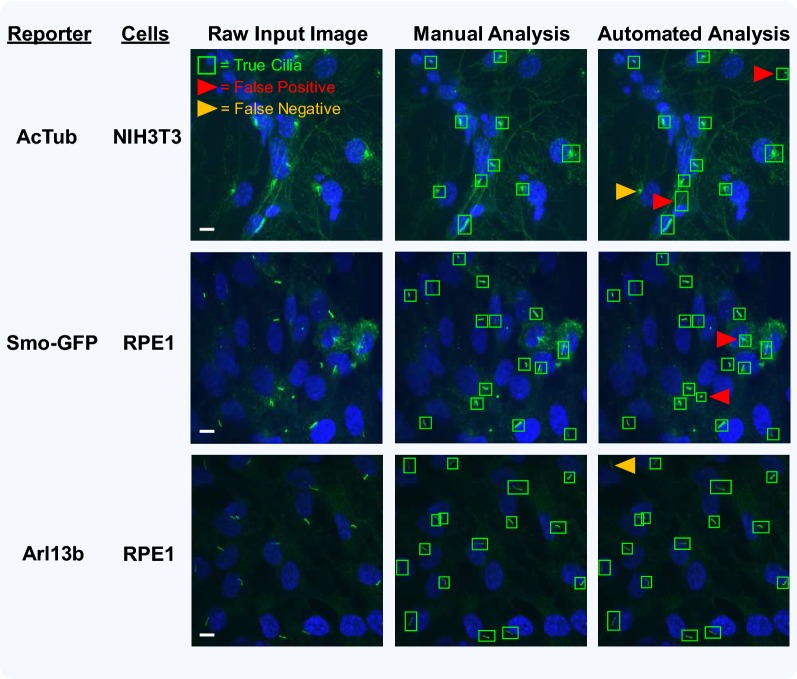
Identification of false-positive and false-negative candidate cilia during automated analysis with ACDC software. In order to evaluate how the rates of false positives (FPs) and false negatives (FNs) change across conditions, two different cell types (NIH3T3 and htert-RPE1) and three different ciliary reporters (AcTub, Smo-GFP, Arl13b) were analyzed. *Left column*: examples of raw input images. *Middle column*: the software’s “full manual analysis” mode was used to detect and accept every candidate based on user criteria. *Right column*: the software’s “automated analysis” mode was used to detect cilia. As shown, most of the manually selected true cilia were detected. However, there were occasionally some FPs (red arrowheads) and FNs (orange arrowheads). Note: for these representative images, FPs and FNs were identified manually (i.e., during semi-automated analysis). Images were acquired with a ×60 (1.4 NA) magnification objective. Scale bars = 10 μm

To assess the accuracy of the software’s analysis, we looked at three cilia reporters (AcTub, Smo-GFP, Arl13b) across two different cell lines (NIH3T3, RPE-1). The analysis results of several of these combinations are shown in Fig. 5, with raw input images (left column), marked-up images from manual analysis (center column), and marked-up images from automated analysis (right column). Although most true cilia appear to be visibly detected, automated detection occasionally gives FPs (indicated by red arrowheads) and FNs (indicated by orange arrowheads). The rate of FPs and FNs could vary depending upon the cellular and imaging conditions such as cell type and cilia reporter. In fully automated analysis mode, the software appeared to perform differently for different reporters, with Arl13b appearing to have fewer FPs and FNs, relative to other reporters such as AcTub and Smo-GFP.

### Quantification of false-positive/negative rates and accuracy scores across different experimental conditions

**Fig. 6:**
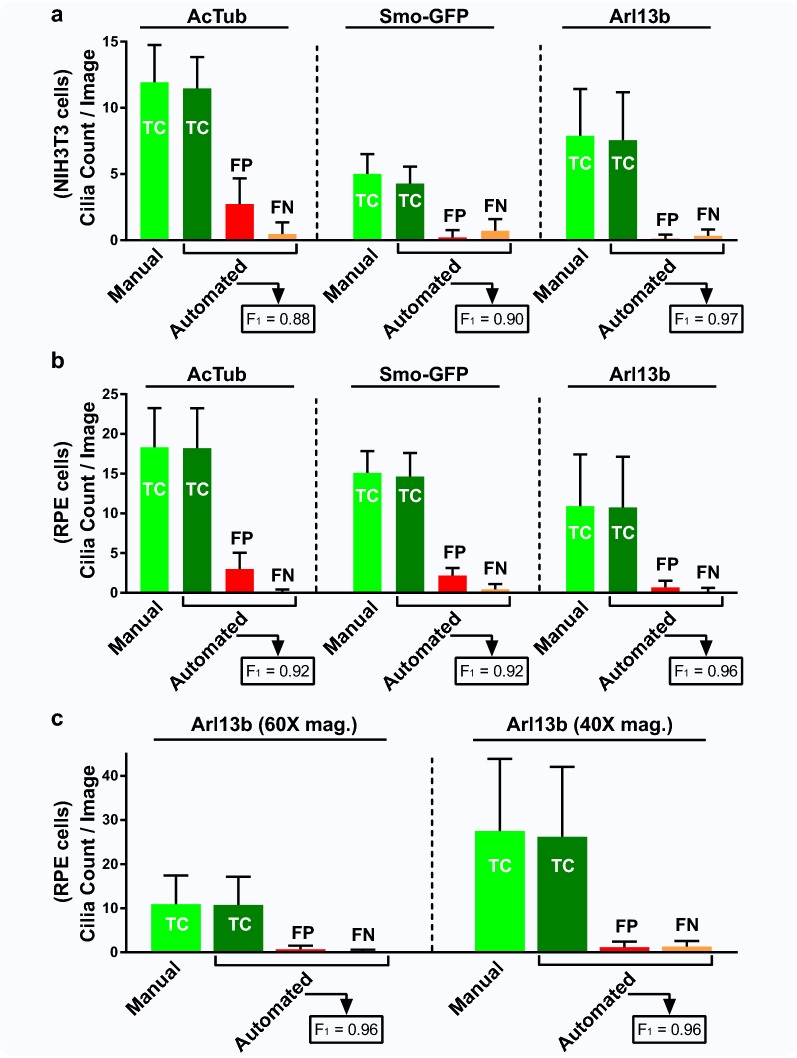
Quantitative analysis of ACDC accuracy in detecting cilia across different experimental conditions. **a** Manual and automated detection of NIH3T3 primary cilia labeled with AcTub, Smo-GFP, and Arl13b. Manual true candidates (TC) represent the total number of true cilia. Automated TC represents the number of manual TC that was detected in automated analysis mode. Among the three reporters, Arl13b had the lowest FP rate (1%) and shared the lowest FN rate with AcTub (4%), resulting in the highest F1 score (F1 = 0.97). Automatically detected TC (dark green bar) plus the corresponding FNs (orange bar) equals the same count as Manual TC (light green bar). **b** Manual and automated detection of htert-RPE1 primary cilia labeled with AcTub, Smo-GFP, and Arl13b. Among the three reporters, Arl13b had the lowest FP rate (6%) and shared the lowest FN rate with AcTub (1%), resulting in the highest F1 score (F1 = 0.96). **c** Manual and automated detection of Arl13b-labeled primary cilia in htert-RPE1 cells at two different image magnifications (×60 vs. ×40). Precision and recall values were similar across both groups, and the F1 scores were identical for images taken at both magnifications (F1 = 0.96). Each condition (i.e., AcTub-labeled NIH3T3 cilia, Arl13b-labeled RPE cilia) was its own separate experiment that was conducted once, so as to demonstrate the cilia FP and FN rates of each condition. The image analysis process for the data set of each condition was repeated three times and each iteration yielded the same values for FP and FN rates. All main bars and error bars are reported as average absolute cilia count per image ± standard deviation per image

Our goal was to see how similarly automated detection performed to manual detection and determine the FP and FN rates of automated analysis across cell types, cilia reporters, and image acquisition magnifications (Fig. 6; Additional file 2: Table S2). Thus, we analyzed 9–25 images (up to 300 cilia) from each condition in Fig. 5 and images of other conditions as well. In Fig. 6a and Additional file 2: Table S2A, the ACDC software was used to manually, automatedly, or semi-automatically detect primary cilia in both NIH3T3 cells stably expressing Smo-GFP and wild-type NIH3T3 cells labeled with antibodies against either AcTub or Arl13b. Cells labeled with AcTub, Smo-GFP, and Arl13b were from separate experiments. For clarity, we graphed the cilia count per image on the y-axis, as opposed percent (%) cilia, because primary cilia—not nuclei—contribute to FNs and largely to FPs. Of note, manual and semi-automated analysis always yielded the same cilia frequency after correcting for FPs and FNs during automated analysis. Automated detected TC (dark green bar) plus the corresponding FNs (orange bar) equals the manual TC (light green bar). Among the three reporters, AcTub had the highest average FP rate on a per-image basis (2.7 ± 1.9 cilia), which is 23% of the manually detected 11.9 ± 2.8 cilia per image. Thus, for every 11.9 AcTub-labeled cilia, 2.7 *additional* non-cilia objects will be detected and treated as true cilia. As expected, AcTub had the lowest precision score (*α* = 0.81). Smo-GFP had the highest average FN rate on a per-image basis (0.7 ± 0.9 or 14% of the manually detected 5.0 ± 1.5 cilia). This means that for every 5.0 true cilia, 0.7 of those true cilia will not be detected. Consequently, Smo-GFP had the lowest recall score (*β* = 0.86). Cilia labeled with Arl13b had the lowest FP and FN rates (1% and 4%) and thus had the highest F1 score (F1 = 0.97), demonstrating that the software’s performance was most accurate when cilia were labeled with Arl13b. Next, we looked at the same three cilia reporters in RPE cells (Fig. 6b; Additional file 2: Table S2B). Among the three reporters, AcTub had the highest average FP rate on a per-image basis (3.0 ± 2.1 cilia), which is 16% of the manually detected 18.3 ± 4.9 cilia per image. This means that for every 18.3 AcTub-labeled RPE cilia, 3.0 non-cilia objects will be detected and treated as true cilia. Consequently, AcTub had the lowest precision score (*α* = 0.86). However, Smo-GFP had nearly similar FP and FN rates as AcTub, which explains why they had similar precision, recall, and F1 scores. Interestingly, Smo-GFP in RPE cells yielded more FPs and fewer FNs compared to Smo-GFP in NIH3T3 cells, indicating detection variability between cell types. In agreement with Fig. 6a, the software’s performance was most accurate in RPE cells when cilia were labeled with Arl13b, given its low FP and FN rates (6% and 1%, respectively) and high F1 score (F1 = 0.96). Lastly, we sought to assess the software’s automated performance across image magnifications (Fig. 6c; Additional file 2: Table S2C). Images from the same experiment of RPE cells with Arl13b cilia staining were taken at 60× magnification (25 images) and 40× magnification (191 images) and analyzed manually, automatedly, and semi-automatedly. Naturally, more cilia were detected with lower magnification (10.9 cilia per 60× image vs. 27.5 cilia per 40× image). We observed comparable FP and FN rates between 60× and 40× magnification (FP: 6% vs. 4%; FN: 1% vs. 5%). The F1 score for both magnifications was F1 = 0.96. We conclude that the software’s automated performance is equally robust and accurate across these two image magnifications. For further detail of Fig. 6, see Additional file 2: Table S2.

### Assessing variability of cilia count, nuclei count, and cilia frequency across separate experiments, different Ground Truths within the same experiment, and different cell confluency levels

Next we wanted to determine how the FP and FN rates for automated cilia and nuclei detection varied: (i) across two different experiments of the same condition and (ii) within the same experiment but using different representative images for detection auto-optimization (Additional file 3: Fig. S3; Additional file 4: Fig. S4). Thus, we analyzed images of RPE cells with Arl13b-stained cilia and DAPI-stained nuclei from two separate experiments, described here as Experiment 1 and Experiment 2 (Additional file 3: Fig. S3A). The images for Experiment 1 were the same images that were analyzed for the RPE Arl13b cilia condition in Fig. 6b. We also tested the performance of the ACDC software on Experiment 2 when using two different images from Experiment 2 for detection auto-optimization. The two representative images from Experiment 2, along with their parameter threshold values after auto-optimization, are shown in Additional file 3: Fig. S3B. Manual and automated software analysis of images from Experiment 1 and Experiment 2 showed that the FP rates, FN rates, and F1 scores were similar across the separate experiments (Additional file 3: Fig. S3C, D). Furthermore, using two different images from Experiment 2 for cilia detection auto-optimization yielded similar FP and FN rates (1–3%) and identical F1 scores (F1 = 0.98). Extending our work with Experiment 2, we demonstrate that the software can detect and trace nuclei in images (Additional file 4: Fig. S4A). In regards to nuclei detection, repeating the automated analysis of the Experiment 2 data set using two different representative images yielded identical FP and FN rates (0.9%) and identical F1 scores (F1 = 0.99) (Additional file 4: Fig. S4B, C). This is due to the fact that the ACDC software segments and detects nuclei irrespective of the representative image used for auto-optimization of cilia detection. After demonstrating the software’s ability to detect cilia and nuclei, we show that these data can be used to calculate cilia frequency (Additional file 4: Fig. S4D). Cilia frequency values for Experiment 2 were calculated by dividing the cilia count per-image values by the nuclei count per-image values. Alternatively, the software conveniently reports cilia frequency for each image in the exported excel file after the analysis ends. Manual analysis of the Experiment 2 data set yielded 72 ± 7% ciliated cells. Automated analysis with detection auto-optimization based on representative image #1 yielded 69 ± 8% ciliated cells. Automated analysis with detection auto-optimization based on representative image #2 yielded 71 ± 9% ciliated cells. We conclude that the software’s automated performance is equally robust and accurate across different experiments of the same condition—in this case with RPE cells with Arl13b-labeled cilia—and also within the same experiment (Experiment 2) but using two different representative images for detection auto-optimization.

We also assessed the software’s performance in two separate experiments in which RPE cells were fixed and stained against Arl13b and DAPI at low and high cell confluency levels to see if there was an effect on cilia frequency measurements (Additional file 5: Fig. S5). The images for both experiments are shown in Additional file 5: Fig. S5A. Low-confluency images possessed, on average, 5.9 ± 2.2 cilia per image, while high-confluency images possessed, on average, 15.7 ± 6.4 cilia per image. For both confluency levels, FP and FN rates for automated cilia detection remained between 1 and 4%, and F1 scores were nearly identical (low confluency, F1 = 0.98 vs. high confluency, F1 = 0.97) (Additional file 5: Fig. S5B, C). Low-confluency images possessed, on average, 10.4 ± 4.2 nuclei per image, while high-confluency images possessed, on average, 39.6 ± 6.4 nuclei per image. In regards to nuclei detection, low-confluency images tended to yield low FN rates (1%) but larger FP rates (4.5%) (Additional file 5: Fig. S5D, E). This means that for every 10.4 nuclei, 0.10 *actual* true nuclei will not be detected during automated analysis, and 0.47 *additional* non-nuclei objects will be detected and treated as nuclei. Together, the data show that low-confluency images were accurately detected by automated analysis, as demonstrated by the high F1 score (F1 = 0.97). Interestingly, high cell confluency images yielded no FPs at all (FP rate = 0%), but yielded a high FN rate (12%). This means that for every 39.6 nuclei, 4.8 *actual* true nuclei will not be detected during automated analysis (Additional file 5: Fig. S5D, E). We attribute this large FN rate of high-confluency images to the phenomenon of more overlapping nuclei. In these events, the software will sometimes treat two overlapping nuclei as a single nucleus. The large FN rate of 12% for nuclei detection in high-confluency images yielded a worse accuracy score (F1 = 0.94) for nuclei detection relative to that of low-confluency images. Lastly, we looked at cilia frequency and confluency (Additional file 5: Fig. S5F). Cilia frequency remained nearly identical between manual and automated analysis for low-confluency images (manual, 59 ± 12% vs. automated, 58 ± 13%), but cilia frequency values varied more between manual and automated analysis for high-confluency images (manual, 39 ± 12% vs. automated, 45 ± 17%). This difference is mainly due to the disparity in nuclei detection between manual and automated analysis for high-confluency images. Thus, we conclude that at a high cell confluency, automated detection of primary cilia remains accurate, but automated detection of nuclei becomes less accurate due to the presence of more overlapping, indistinguishable nuclei, which can obscure subsequent cilia frequency calculations. We therefore recommend using images in which cell confluency is not extremely high.

### Quantitative assessment of automated cilia length analysis and speed enhancement

**Fig. 7:**
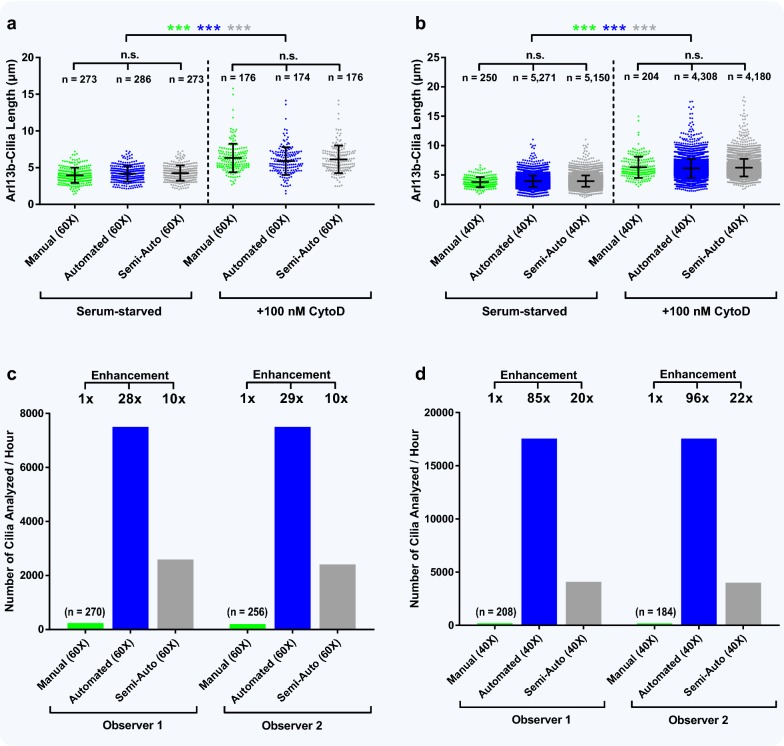
ACDC accurately measures cilia length and provides a major speed enhancement in cilia analysis. **a** htert-RPE1 cells were grown in serum-containing media and then in serum starvation media for 2 days supplemented with or without 100 nM cytochalasin D (CytoD) for the last 8–16 h. Cells were stained against Arl13b and DAPI and images were captured at 60× magnification. Images were acquired on the same day from the same experiment. For both conditions, the ACDC software was used to manually, automatedly, and semi-automatically measure primary cilia length from 25 images. All analysis modes showed that CytoD-treated cilia were significantly longer than cilia that were just serum-starved. There was no significant (n.s.) difference in length between analysis modes within each condition. Cilia count is shown above each data series. For both serum starvation and +CytoD conditions, 10–25 images were used for analysis. **b** Images of ciliated cells were taken at ×40 magnification (mag.) and then analyzed. Similar mean lengths were observed between ×60-mag. and ×40-mag. serum-starved conditions and also between ×60-mag. and ×40-mag. +CytoD conditions. All analysis modes showed that CytoD-treated cilia were significantly longer than cilia that were just serum-starved. There was no significant (n.s.) difference in length between analysis modes within each condition. Scatter plots are represented as mean length ± standard deviation from one experiment. Statistical significance in which *p* < 0.001 is represented by (***). Cilia count is shown above each data series. For both serum starvation and +CytoD conditions, 5 images were analyzed by ACDC manual analysis and 190–200 images were analyzed by automated and semi-automated analysis. **c, d** Cilia analysis rate (number of cilia analyzed/hour) for manual, automated, and semi-automated analyses of the ×60-mag. and ×40-mag. serum condition images from (**a**) and (**b**), respectively. Speed enhancement, relative to ACDC manual analysis, is shown at the top of each graph

In addition to accurately detecting nuclei and cilia, the ACDC software measures cilia length. Thus, after determining the FP and FN rates across various conditions, we aimed to assess the software’s performance for cilia length (Fig. 7). We used images of RPE cells with ciliary Arl13b staining because of the software’s high precision, recall, and F1 scores associated with Arl13b as a cilia reporter. In Fig. 7a, we analyzed two different groups of RPE cells from the same experiment—one grown in serum starvation media and one grown in serum starvation media with overnight supplementation of cytochalasin D (CytoD), an actin depolymerizing agent that induces ciliogenesis. Images of both culture conditions were taken on the same day at 60× magnification and analyzed manually, automatedly, and semi-automatedly. Within treatment conditions, mean length measurements were considerably consistent, as no statistically significant differences were observed (serum starvation: manual = 3.96 ± 1.02 μm, auto = 4.18 ± 1.06 μm, semi-auto = 4.26 ± 1.04 μm; CytoD treatment: manual = 6.32 ± 1.93 μm, auto = 5.90 ± 1.88 μm, semi-auto = 6.13 ± 1.86 μm). However, when comparing mean lengths between treatment conditions by manual, automated, and semi-automated analysis, statistical significance (*p* < 0.001) was observed across all three detection modes. Similar results were observed for images of ciliated cells taken at 40× magnification (serum starvation: manual = 3.79 ± 0.84 μm, auto = 3.95 ± 0.95 μm, semi-auto = 3.97 ± 0.96 μm; CytoD treatment: manual = 6.32 ± 1.81 μm, auto = 6.12 ± 1.60 μm, semi-auto = 6.26 ± 1.50 μm) (Fig. 7b). Thus, by comparing the manual, automated, and semi-automated analysis results with each other within treatment groups, we showed that the ACDC software, even with the presence of some FPs and FNs, can accurately report cilia length. Notably, the automated and semi-automated analyses of the 40× images allowed for a more thorough study of large populations of cells, as nearly 200 images (*n* > 5000 cilia) were imported and rapidly analyzed.

The major benefit of the ACDC software is the streamlined analysis time (Fig. 7c, d). For the images taken at 60× magnification, automated analysis was 28× to 29× faster than manual analysis, and semi-automated analysis was 10× faster than manual analysis (Fig. 7c). Each 60× image required, on average, 5.2 s to automatically analyze everything without corrections (Additional file 6: Table S6). For images taken at 40× magnification, automated analysis was 85× to 96× faster than manual analysis, and semi-automated analysis was 20× to 22× faster than manual analysis (Fig. 7d). Each 40× image required, on average, 5.6 s to automatically analyze everything without corrections (Additional file 6: Table S6). The graphs in Fig. 7c, d correspond to the analyses of the images of serum-starved cells without CytoD treatment in Fig. 7a, b, respectively; analysis times for 60× and 40× images of CytoD-treated cells were nearly identical to those reported in Fig. 7c, d. To report cilia analysis rates for automated and semi-automated analyses shown in Fig. 7c, d, we recorded the time required to measure every cilium from the imported data sets of Fig. 7a, b, respectively, and then extrapolated those analysis rates to a per-hour basis. The difference in speed enhancements between 60× and 40× images can be explained by the fact that 60× images possess an average of 10.9 cilia per image while 40× images possess an average of 27.5 cilia per image. More nuclei and more cilia will require several more milliseconds of automated analysis. As previously stated, the quality of data from semi-automated analysis is naturally as good as that from full manual analysis, but with the benefit of spending only 5–10% of the time required for manual analysis. Thus, in these examples the software offers large time-savings while losing none of the robustness and accuracy of standard manual analysis. Of note, the time required to manually analyze nuclei and cilia using the ACDC software was similar to that of other manual analysis approaches (e.g., using ImageJ’s segmented line tool) (Additional file 7: Fig. S7). Instead of showing analysis time on the y-axis, we show cilia analysis rate, adjusted to a per-hour basis. From these data, we believe our comparisons in this study to ACDC manual analysis are reflective of currently used analysis methodologies.

**Fig. 8:**
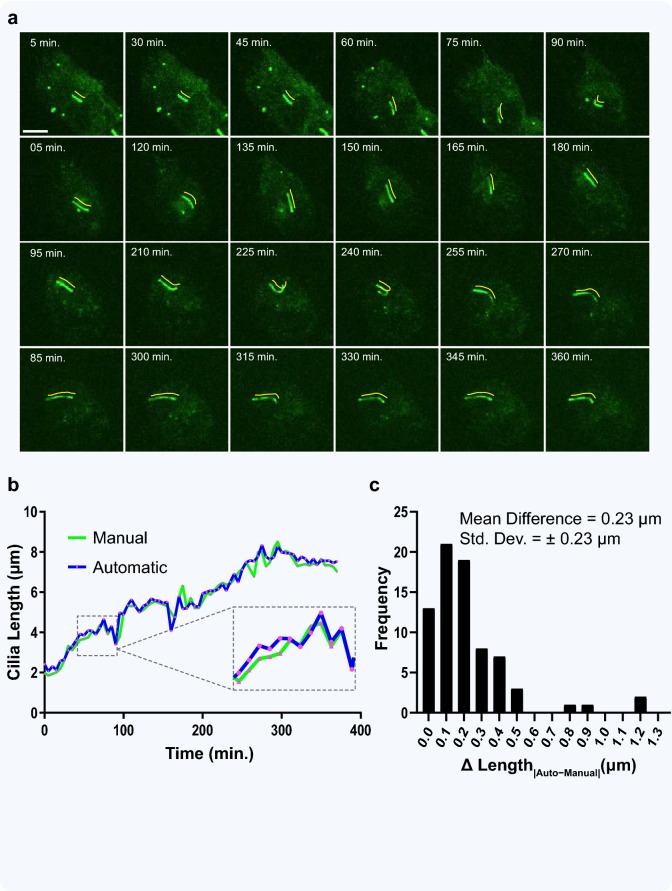
Application of ACDC to automated analysis of cilia movies. **a** A gallery of images from a live-cell spinning-disk confocal microscopy movie (370 min, 60× magnification) of a single cilium from an htert-RPE1 cell stably expressing Smoothened-pHluorin GFP, serum-starved and treated with 100 nM CytoD to promote ciliogenesis. Image frames of the GFP channel were taken from every 5 min and analyzed with the ACDC software. Yellow lines (shifted 10 pixels up and 10 pixels right) show the cilia ‘spline’ generated by the software during automated length measurement. Scale bar = 5 μm. **b** Movie frames were both manually and automatically analyzed for cilia length (0.143 μm/pixel). Line graph shows cilium growth over time; pink data points on both lines represent the analyzed frames. **c** Histogram of length measurement differences between manual and automated analysis reveals only a nominal difference of less than 2 pixels

To show the value of this application in a proof of concept case study, we looked at measuring cilia length over an extended period of time (Fig. 8). A 370-min movie of CytoD-treated RPE-Smo-GFP cells was obtained by live-cell confocal imaging at 60× magnification. Image frames of the GFP channel were taken from every 5 min of the movie and analyzed using the ACDC software (Fig. 8a). Despite changes over time in cilium position, length, intensity, and curvature, the software’s automated length measurements were nearly identical to those of its manual length measurements in each frame (Fig. 8b). A histogram of length differences between the two analysis modes shows a mean difference of 0.23 μm ± 0.23 μm (Fig. 8c). Given that the setting for 60× images is 1 pixel = 0.143 μm, this translates to less than a 2-pixel discrepancy between manual and automated analysis. Further, when the few extreme outliers are corrected for by semi-automated analysis, the mean length difference decreases to 0.18 μm ± 0.14 μm.

## Discussion

Quantifying cilia frequency and length is typically done by time-consuming manual analysis, yet there are no image analysis programs developed specifically for the automated detection of primary cilia. In this work, we present the ACDC software as a viable solution. First, we demonstrated the software’s versatility and robustness by successfully detecting primary cilia across different reporters and cell types (Figs. 5, 6a, b; Additional file 2: Table S2A, B). While many cilia reporters work with ACDC, Arl13b had the lowest FP and FN rates. We also looked at different image magnifications and found that Arl13b-labeled cilia from images taken at 60× magnification were just as accurately detected (F1 score = 0.96) as Arl13b-labeled cilia from images taken at 40× magnification (Fig. 6c; Additional file 2: Table S2C). This is beneficial for cilia researchers, as 40× images typically possess more cilia than do 60× images. Next, we demonstrated that the ACDC software measured similar FP and FN rates, for both cilia and nuclei detection, across two different experiments of the same condition (RPE cells with Arl13b-labeled cilia) and within the same experiment but using different representative images for detection auto-optimization (Additional file 3: Fig. S3; Additional file 4: Fig. S4). In doing so, we showed that the software could detect cilia and nuclei and subsequently calculate cilia frequency. Furthermore, we showed that very high cell confluency can possibly obscure cilia frequency calculations, as the nuclei count for high-confluency images tended to possess more false negatives (FN rate = 12%) compared to low-confluency images (Additional file 5: Fig. S5). We therefore recommend using images in which cell confluency is not extremely high. Then, we demonstrated as a proof of concept the software’s functionality in two biological contexts: (i) accurately measuring statistically significant changes in cilia length after drug treatment (Fig. 7a, b), and (ii) monitoring cilia length from 75 individual movie frames, which yielded an average length difference of 1–2 pixels compared to manual analysis (Fig. 8). Importantly, we demonstrated the remarkable time-savings offered by the software (Fig. 7c, d). Nearly 200 images (*n* > 5000 cilia) taken at 40× magnification were analyzed in one session, yielding a consistent analysis time of 5.6 s per image. This translated to almost a 100-fold enhancement in analysis time compared to the software’s manual analysis time of the same data set, which required around 476 s per 40× image (Additional file 6: Table S6). Thus, by automated analysis, nearly 18,000 cilia could be analyzed per hour, depending on the confluency of cells at the time of fixation. Even semi-automated analysis, in which FPs and FNs were corrected, yielded a 20- to 22-fold enhancement compared to manual analysis of the same data set (Fig. 7d).

The notable performance of the software stems partly from its pre-processing algorithm. While we utilize standard equalization and smoothing operations seen in other studies [41–43], we also employ an enhanced binarizations step with a Gaussian filter to better account for the dynamic intensity variations of different cilia candidates (Fig. 2). The robust performance of the software also stems from its detection auto-optimization ability, based on the four previously discussed parameters (Fig. 3). With over 5.5 million parameter combinations to choose from, automated detection of candidates can be optimized despite vastly differing experimental conditions. We believe this auto-optimization feature also reduces the potential for user bias, as the predominant source of bias would originate only from manually choosing the representative image for the data set. Indeed, in this study, we observed low error rates associated with the software’s automated analysis mode (Fig. 6). Other studies that have conducted screens with automated detection programs such as IN Cell Analyzer 2000 Imaging system and IN Cell Developer software (GE Healthcare) have not reported associated error rates [10]. Yet in these types of studies, significant error could arise in the form of FPs, wherein numerous secreted ciliary vesicles are interpreted as actual cilia by the detection software [44, 45]. Other high-throughput screens have used the ‘GPCR Segmentation’ algorithm of CytoShop HCS analysis software for cilia detection [11], but had to manually adjust threshold values for multiple parameters such as object scale for aggregate cilia detection and minimum intensity peak height. A process like this introduces potential selection bias, and the ACDC software attempts to mitigate this possibility by optimizing detections parameters based on a comprehensive representative image. Therefore, we recommend choosing a representative image with a cilia count close to that of the data set’s estimated per-image average, as well as an image with some examples of cilia varying in shape and intensity (depending on the cilia reporter). Once the representative image is chosen, the user must establish the Ground Truth by manually selecting the candidates they consider actual, true cilia (Fig. 4 iii). We believe selection bias is essentially non-existent here, as cilia stained against AcTub, Smo-GFP, and Arl13b seem to be distinguishable from other objects such as fixation artifacts, random aggregates, cytoplasmic projections, mitotic structures, and secreted vesicles [5, 33, 46, 47]. Thus, choosing the representative image itself, but not establishing the Ground Truth from that image, stands as the predominant source for detection bias.

Ideally, this software would be used for fully automated analysis, in which case the FP and FN rates need to be acceptable to the user. Semi-automated analysis is also a viable option, as it guarantees the accuracy of manual analysis at the cost of extra time to correct for detection errors. In either method, we recommend using Arl13b as a cilia reporter, as Arl13b-labeled cilia were most accurately detected across all experimental conditions (Fig. 6). However, we do not discourage using AcTub and Smo-GFP as reporters, as they still yielded high F1 scores comparable to that of Arl13b. AcTub had lower F1 scores due to the presence of more FPs from cytosolic structures and mitotic events that also possessed bright tubulin labeling. Smo-GFP had lower F1 scores due to the presence of more FPs from plasma membrane Smo-GFP and due to the presence of more FNs from variable Smo expression among cilia (very dim cilia that were overlooked during detection).

Herein, we have demonstrated the applicability of this developed approach and have essentially established the basis for more extensive, high-throughput studies. Additionally, this software could be modified and implemented in other image analysis platforms, and our validation data from this study could be used to test the equivalency of such an approach. In the future, we plan on evaluating the software’s detection accuracy for cilia labeled with other reporters such as INPP5E, IFT88, SSTR3, 5HT6, MCHR1, and ACIII [48–53]. Eventually, we also plan on using the software to analyze videos containing multiple cilia by developing a cilium-tracking algorithm. Lastly, we hope to extend this software to the analysis of 3D images of ciliated cells. In doing so, we would consider the different methods for length analysis evaluated by Dummer et al. [54] and Saggesse et al. [55], including maximum intensity projection, Pythagorean theorem, 3D alternative angled slicing, and reconstruction of each cilium through processes of deconvolution and Gaussian blurring.

## Conclusions

Studying properties of primary cilia such as frequency and length is important for understanding multiple complex human diseases. However, current methods for primary cilia image analysis are time-consuming and prone to selection bias. We developed a new image analysis software, ACDC, specifically for the automated detection and analysis of images of ciliated cells. The software is robust and accurate, and can offer nearly two orders of magnitude in time-savings compared to conventional manual analysis. Future versions of ACDC could presumably be extended to 3D image analysis and screening assays.

## Additional files

**Additional file 1: Figure S1.** Surface plots of F1 scores for different cilia reporters. Although the F1 score is ultimately based on four parameters, here we depict a 3D surface plot with two of the parameters on the x- and y-axes and F1 score on the z-axis for visualization purposes. Each plot is based on the corresponding image of ciliated RPE cells. F1 score values for automated analysis of each image were calculated by incrementally increasing one parameter threshold while holding the other parameter threshold constant and then counting the number of FPs and FNs. These plots illustrate that there could be many parameter combinations that results in the same maximal F1 score, in which case the program’s default is to choose the parameter combination that minimizes all parameter values while retaining that maximal F1 score. Of the three cilia reporters, Arl13b seems to be the ‘best’ reporter, as its surface plot has the largest area at F1 = 1.00. Thus, at most combinations of SNR and STD threshold values, all Arl13b-labeled true cilia will be detected and no FPs will be included.

**Additional file 2: Table S2.** Tabular data of Fig. 6. For each group (e.g. AcTub-labeled cilia in NIH3T3 cells, etc.), absolute cilia count from analyzed images were averaged and reported as manual true cilia (“Manual TC”) averages ± standard deviations. Thus, a data set with a Manual TC of 11.9 ± 2.8 indicates an average of 11.9 cilia per image with a standard deviation of 2.8 cilia per image. “Automated TC” average counts differ from those of “Manual TC” because in automated analysis mode some true cilia might not be detected by the software. An “Automated TC” of 11.4 ± 2.4 (96% of “Manual TC”) indicates that, on average, the software auto-detects 96% of true cilia in each image. A false positive (FP) rate of 2.7 ± 1.9 (23% of “Manual TC”) indicates that, on average, the software will include 2.7 *additional* objects (23% more fake cilia) in each image, on top of the 11.4 ± 2.4 (96%) detected true cilia per image. A false negative (FN) rate of 0.5 ± 0.9 (4% of “Manual TC”) indicates that, on average, the software will not detect 4% of the “Manual TC” in each image. Precision (*α*) is calculated using the data from the “Automated TC” and “FP” rows. Recall (*β*) is calculated using the data from the “Automated TC” and “FN” rows. F1 score is calculated using the data from the “Precision (*α*)” and “Recall (*β*)” rows. Accuracy ratings are based on F1 score values, which range from 0 to 1.00 (0.95–1.00, ++++ ; 0.90–0.94, +++; 0.85–0.89, ++/+++).

**Additional file 3: Figure S3.** Ciliary FP/FN rates across separate experiments or different Ground Truths within the same experiment. (A) These are the analyzed images of RPE cells with Arl13b-stained cilia and DAPI-stained nuclei from two separate experiments (Experiment 1 and Experiment 2). The images for Experiment 1 were the same images that were analyzed for the RPE Arl13b cilia condition in Fig. 6b. (B) The images from Experiment 2 were analyzed two times using two different representative images to establish two different Ground Truths. The two different representative images from the Experiment 2 data set are shown, and below are their respective parameter threshold values after detection auto-optimization. (C) Manual and automated detection of RPE primary cilia labeled with Arl13b in 60×-mag. images. Manual true candidates (TC) represent the total number of true cilia (light green bar). Automated TC represents the number of manual TC that was detected in automated analysis mode (dark green bar). Across different experiments (i.e. Experiment 1 vs. Experiment 2), the FP rates, FN rates, and F1 scores were similar. When repeating the same experiment (i.e. Experiment 2), but using different representative images of the data set for detection auto-optimization, FP and FN rates were similar (1–3%) and F1 scores were identical (F1 = 0.98). (D) Tabular data for the values depicted in (C). Accuracy ratings are based on F1 score values, which range from 0 to 1.00 (0.95–1.00, ++++ ; 0.90–0.94, +++; 0.85–0.89, ++/+++). All measurements are reported as averages ± standard deviations of multiple images from one experiment.

**Additional file 4: Figure S4.** Quantitative analysis of ACDC accuracy in detecting nuclei and cilia frequency. (A) Examples of nuclei detection with ACDC software of the two representative images used for Experiment 2. (B) Manual and automated detection of RPE nuclei labeled with DAPI in 60×-mag. images. Manually-detected nuclei represent the total number of true nuclei (light green bar). Automatedly-detected nuclei represent the proportion of true nuclei that were detected in automated analysis mode (dark green bar). In regards to nuclei detection, repeating the analysis using two different representative images from the same Experiment 2 data set yielded identical FP and FN rates (0.9%) and identical F1 scores (F1 = 0.99). Note that detection auto-optimization from representative images is not utilized for nuclei segmentation and detection. (C) Tabular data for the values depicted in (B). Accuracy ratings are based on F1 score values, which range from 0 to 1.00 (0.95–1.00, ++++ ; 0.90–0.94, +++; 0.85–0.89, ++/+++). (D) Manual and automated analysis of cilia frequency for the images from Experiment 2. Cilia frequency values were calculated by dividing the cilia count/image values by the nuclei count/image values. All measurements are reported as averages ± standard deviations of multiple images from one experiment.

**Additional file 5: Figure S5.** High cell confluency can affect automated cilia and nuclei detection and cell frequency measurements. (A) Analyzed images from two separate samples of differing cell confluency (low and high). (B and C) Bar graphs and corresponding tabular data of manual and automated analysis of RPE Arl13b-labeled cilia in 60×-mag. images. Manual true candidates (TC) represent the total number of true cilia (light green bar). Automated TC represents the number of manual TC that was detected in automated analysis mode (dark green bar). Despite differences in cell confluency, FP and FN rates for cilia detection remained between 1 and 4% and F1 scores were similar (low confluency, F1 = 0.98 vs. high confluency, F1 = 0.97). (D and E) Bar graphs and corresponding tabular data of manual and automated analysis of RPE nuclei in the same 60×-mag. images. FP rates for nuclei detection were greater in low confluency images than in high confluency images (4.5% vs. 0%), but FN rates for nuclei detection were much greater in high confluency images than in low confluency images (12% vs. 1%). Consequently, the large FN rate of 12% for nuclei detection in high confluency images yielded a worse accuracy score (F1 = 0.94) compared to that of low confluency images (F1 = 0.97). (F) Manual and automated analysis of cilia frequency for the low and high confluency data sets. Cilia frequency was calculated by dividing the cilia count per image values by the nuclei count per image values. Cilia frequency remained nearly identical between manual and automated analysis for low confluency images, but cilia frequency values varied more between manual and automated analysis for high confluency images (manual, 39% ± 12% vs. automated, 45% ± 17%). This difference is due to the disparity in nuclei detection between manual and automated analysis for high confluency images. All measurements are reported as averages ± standard deviations of multiple images from one experiment.

**Additional file 6: Table S6.** Tabular data of Fig. 7. Images of RPE cells with Arl13b-stained cilia, taken at two different magnifications, were analyzed manually, automatedly, and semi-automaticallywith the ACDC software. Analysis times were recorded for each image and then averaged. Analysis times for fully automated analysis (“Auto) are greatly faster than those of manual analysis. Standard deviations of fully automated analysis times are much smaller than those of semi-automated analysis times (“Semi-Auto”), which are smaller than those of manual analysis times. Analysis times include the time required to count nuclei, count cilia, and measure cilia length. All measurements are reported as averages ± standard deviations of multiple images from one experiment.

**Additional file 7: Figure S7.** ACDC manual analysis versus ImageJ manual analysis. ACDC software’s manual analysis mode and ImageJ’s segmented line tool were used to manually measure microscopy images of Arl13b-labeled ciliated cells taken at 60× magnification. The analysis results of two independent, unbiased observers were compared.

## Data Availability

The ACDC software was developed in MATLAB R2016b (Windows OS), and is available upon request for academic use from the corresponding authors. Similarly, the cilia microscopy data sets are available upon request from the corresponding authors.

## Abbreviations

ACDC: automated cilia detection in cells
SNR: signal-to-noise ratio
STD: standard deviation
TC: true cilia
FP: false positive
FN: false negative
SDCM: spinning-disk confocal microscopy
AcTub: acetylated *α*-tubulin
Smo: smoothened
GFP: green fluorescent protein
PBS: phosphate-buffered saline
RPE: retinal pigment epithelium
