# Supplementary figures and images for: New software for automated cilia detection in cells (ACDC)
Source: Cilia. 2019 Aug 1;8:1. doi: 10.1186/s13630-019-0061-z (PMC6670212; doi:10.1186/s13630-019-0061-z)

**Arl13b**

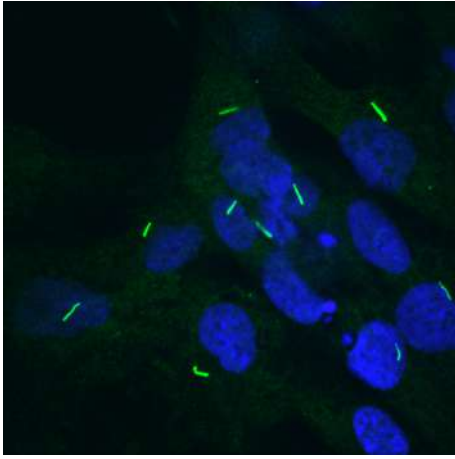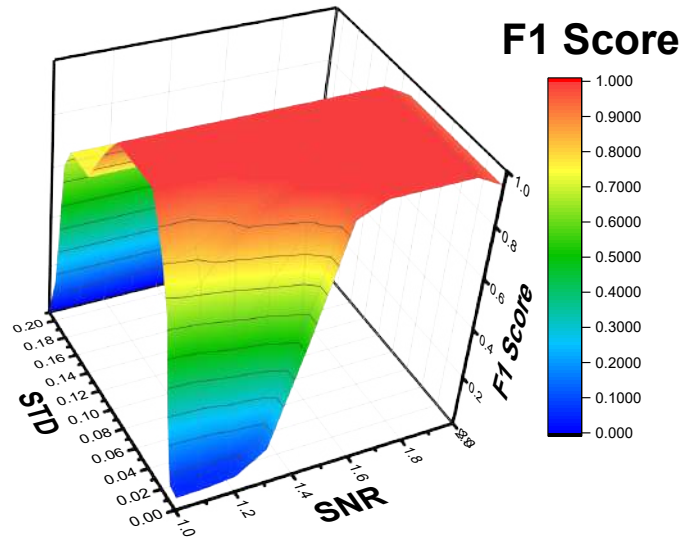

**Smo-GFP**

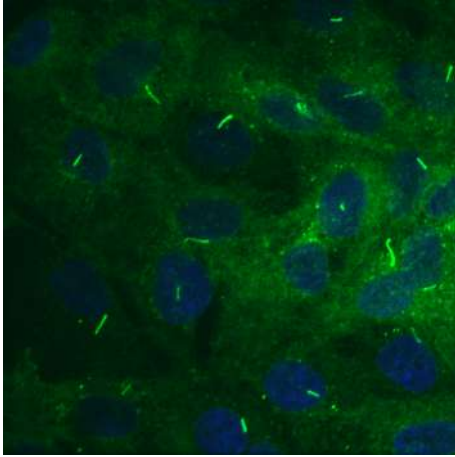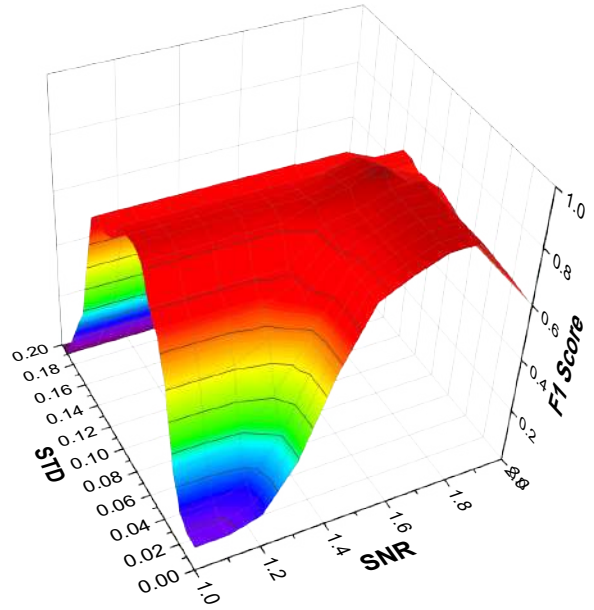

**AcTub**

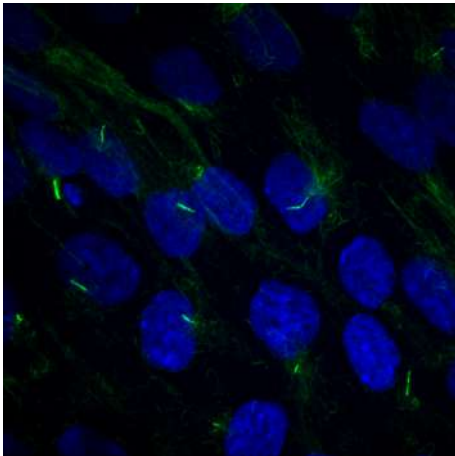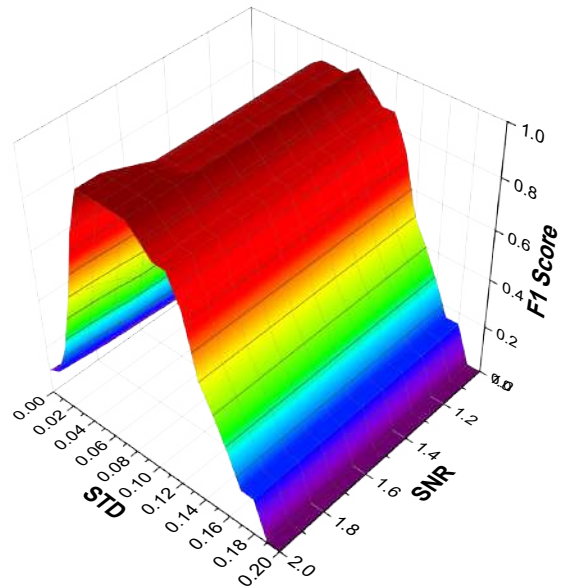

Supplement: Supplementary file 1 — Additional file 1: Figure S1. Surface plots of F1 scores for different cilia reporters. Although the F1 score is ultimately based on four parameters, here we depict a 3D surface plot with two of the parameters on the x- and y-axes and F1 score on the z-axis for visualization purposes. Each plot is based on the corresponding image of ciliated RPE cells. F1 score values for automated analysis of each image were calculated by incrementally increasing one parameter threshold while holding the other parameter threshold constant and then counting the number of FPs and FNs. These plots illustrate that there could be many parameter combinations that results in the same maximal F1 score, in which case the program’s default is to choose the parameter combination that minimizes all parameter values while retaining that maximal F1 score. Of the three cilia reporters, Arl13b seems to be the ‘best’ reporter, as its surface plot has the largest area at F1 = 1.00. Thus, at most combinations of SNR and STD threshold values, all Arl13b-labeled true cilia will be detected and no FPs will be included. [file 13630_2019_61_MOESM1_ESM.pdf]

**A**

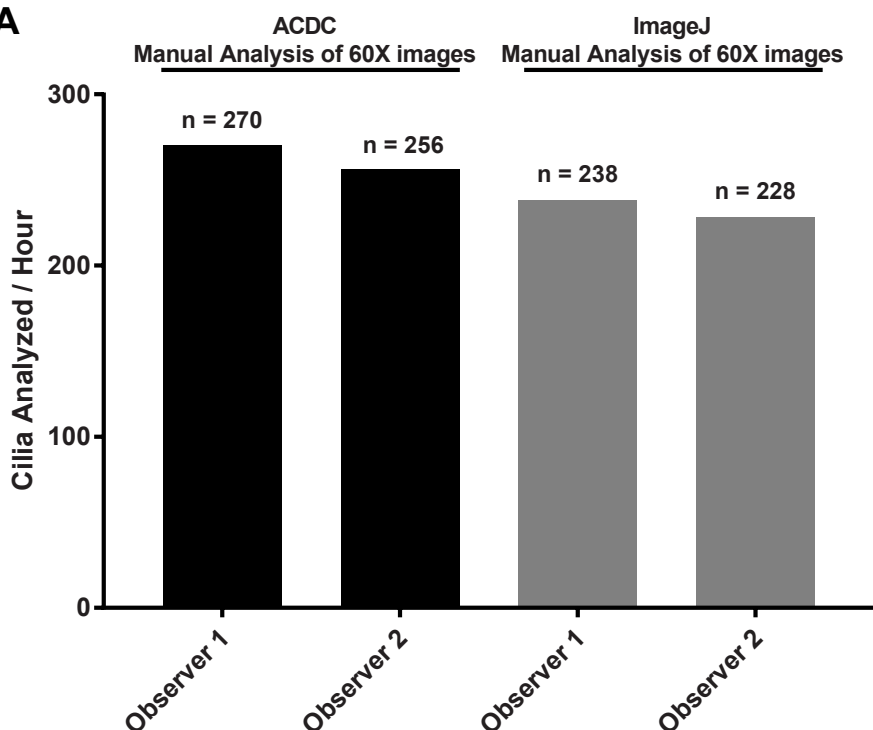

**B**

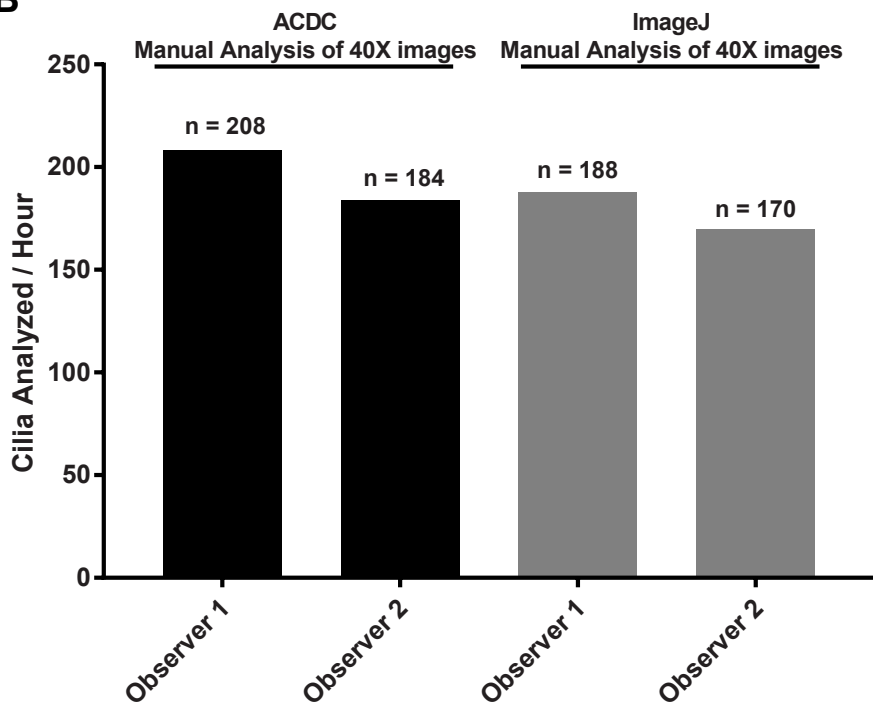

Supplement: Supplementary file 7 — Additional file 7: Figure S7. ACDC manual analysis versus ImageJ manual analysis. ACDC software’s manual analysis mode and ImageJ’s segmented line tool were used to manually measure microscopy images of Arl13b-labeled ciliated cells taken at 60× magnification. The analysis results of two independent, unbiased observers were compared. [file 13630_2019_61_MOESM7_ESM.pdf]
