# Supplementary material for: New software for automated cilia detection in cells (ACDC)
Source: Cilia. 2019 Aug 1;8:1. doi: 10.1186/s13630-019-0061-z (PMC6670212; doi:10.1186/s13630-019-0061-z)
Supplement: Supplementary file 2 — Additional file 2: Table S2. Tabular data of Fig. 6. For each group (e.g. AcTub-labeled cilia in NIH3T3 cells, etc.), absolute cilia count from analyzed images were averaged and reported as manual true cilia (“Manual TC”) averages ± standard deviations. Thus, a data set with a Manual TC of 11.9 ± 2.8 indicates an average of 11.9 cilia per image with a standard deviation of 2.8 cilia per image. “Automated TC” average counts differ from those of “Manual TC” because in automated analysis mode some true cilia might not be detected by the software. An “Automated TC” of 11.4 ± 2.4 (96% of “Manual TC”) indicates that, on average, the software auto-detects 96% of true cilia in each image. A false positive (FP) rate of 2.7 ± 1.9 (23% of “Manual TC”) indicates that, on average, the software will include 2.7 additional objects (23% more fake cilia) in each image, on top of the 11.4 ± 2.4 (96%) detected true cilia per image. A false negative (FN) rate of 0.5 ± 0.9 (4% of “Manual TC”) indicates that, on average, the software will not detect 4% of the “Manual TC” in each image. Precision (α) is calculated using the data from the “Automated TC” and “FP” rows. Recall (β) is calculated using the data from the “Automated TC” and “FN” rows. F1 score is calculated using the data from the “Precision (α)” and “Recall (β)” rows. Accuracy ratings are based on F1 score values, which range from 0 to 1.00 (0.95–1.00, ++++ ; 0.90–0.94, +++; 0.85–0.89, ++/+++). [file 13630_2019_61_MOESM2_ESM.pdf]

A (see Fig. 6A)

**NIH3T3 cells**	AcTub	Smo-GFP	Arl13b
Images Analyzed	15	14	9
Manual TC	11.9±2.8	5.0±1.5	7.9±3.5
Automated TC	11.5±2.4 **(96%)**	4.3±1.3 **(86%)**	7.6±3.6 **(96%)**
FP	2.7±1.9 **(23%)**	0.2±0.6 **(4%)**	0.1±0.3 **(1%)**
FN	0.5±0.9 **(4%)**	0.7±0.9 **(14%)**	0.3±0.5 **(4%)**
Precision (α)	0.81	0.95	0.99
Recall (β)	0.96	0.86	0.96
**F1 score**	**0.88**	**0.90**	**0.97**
Accuracy	++/+++	+++	++++

B (see Fig. 6B)

**RPE cells**	AcTub	Smo-GFP	Arl13b
Images Analyzed	9	11	25
Manual TC	18.3±4.9	15.1±2.7	10.9±6.5
Automated TC	18.2±5.0 **(99%)**	14.6±3.0 **(97%)**	10.8±6.4 **(99%)**
FP	3.0±2.1 **(16%)**	2.2±0.9 **(14%)**	0.7±0.8 **(6%)**
FN	0.1±0.3 **(1%)**	0.5±0.7 **(3%)**	0.2±0.5 **(1%)**
Precision (α)	0.86	0.87	0.94
Recall (β)	0.99	0.97	0.99
**F1 score**	**0.92**	**0.92**	**0.96**
Accuracy	+++	+++	++++

C (see Fig. 6C)

**RPE cells**	Arl13b (60X)	Arl13b (40X)
Images Analyzed	25	191
Manual TC	10.9±6.5	27.5±16.4
Automated TC	10.8±6.4 **(99%)**	26.2±15.9 **(95%)**
FP	0.7±0.8 **(6%)**	1.2±1.3 **(4%)**
FN	0.2±0.5 **(1%)**	1.3±1.3 **(5%)**
Precision (α)	0.94	0.96
Recall (β)	0.99	0.95
**F1 score**	**0.96**	**0.96**
Accuracy	++++	++++
