# Supplementary material for: New software for automated cilia detection in cells (ACDC)
Source: Cilia. 2019 Aug 1;8:1. doi: 10.1186/s13630-019-0061-z (PMC6670212; doi:10.1186/s13630-019-0061-z)
Supplement: Supplementary file 3 — Additional file 3: Figure S3. Ciliary FP/FN rates across separate experiments or different Ground Truths within the same experiment. (A) These are the analyzed images of RPE cells with Arl13b-stained cilia and DAPI-stained nuclei from two separate experiments (Experiment 1 and Experiment 2). The images for Experiment 1 were the same images that were analyzed for the RPE Arl13b cilia condition in Fig. 6b. (B) The images from Experiment 2 were analyzed two times using two different representative images to establish two different Ground Truths. The two different representative images from the Experiment 2 data set are shown, and below are their respective parameter threshold values after detection auto-optimization. (C) Manual and automated detection of RPE primary cilia labeled with Arl13b in 60×-mag. images. Manual true candidates (TC) represent the total number of true cilia (light green bar). Automated TC represents the number of manual TC that was detected in automated analysis mode (dark green bar). Across different experiments (i.e. Experiment 1 vs. Experiment 2), the FP rates, FN rates, and F1 scores were similar. When repeating the same experiment (i.e. Experiment 2), but using different representative images of the data set for detection auto-optimization, FP and FN rates were similar (1–3%) and F1 scores were identical (F1 = 0.98). (D) Tabular data for the values depicted in (C). Accuracy ratings are based on F1 score values, which range from 0 to 1.00 (0.95–1.00, ++++ ; 0.90–0.94, +++; 0.85–0.89, ++/+++). All measurements are reported as averages ± standard deviations of multiple images from one experiment. [file 13630_2019_61_MOESM3_ESM.pdf]

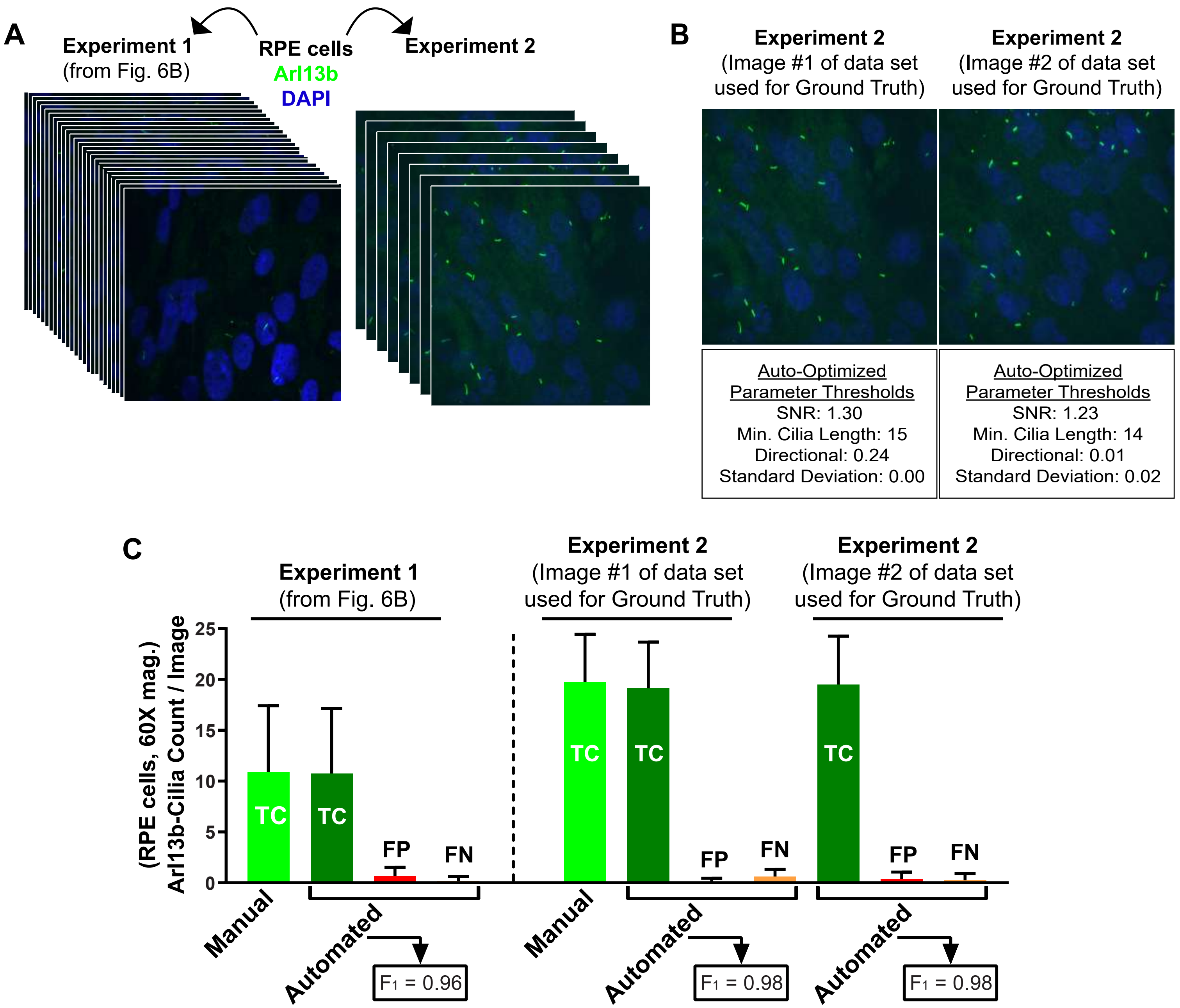

D

RPE cells (Arl13b cilia)	Experiment 1 (From Fig. 6B)	Experiment 2 (Ground Truth #1)	Experiment 2 (Ground Truth #2)
Images Analyzed	25	8	8
Manual TC	10.9±6.5	19.8±4.7	19.8±4.7
Automated TC	10.8±6.4 **(99%)**	19.1±4.5 **(95%)**	19.5±4.8 **(95%)**
FP	0.7±0.8 **(6%)**	0.1±0.3 **(1%)**	0.4±0.7 **(2%)**
FN	0.2±0.5 **(1%)**	0.6±0.7 **(3%)**	0.3±0.7 **(1%)**
Precision (α)	0.94	0.99	0.98
Recall (β)	0.99	0.97	0.99
**F1 score**	**0.96**	**0.98**	**0.98**
Accuracy	++++	++++	++++
