# Supplementary material for: New software for automated cilia detection in cells (ACDC)
Source: Cilia. 2019 Aug 1;8:1. doi: 10.1186/s13630-019-0061-z (PMC6670212; doi:10.1186/s13630-019-0061-z)

A

Example of Nuclei Detection Experiment 2, Image #1

Example of Nuclei Detection Experiment 2, Image #2

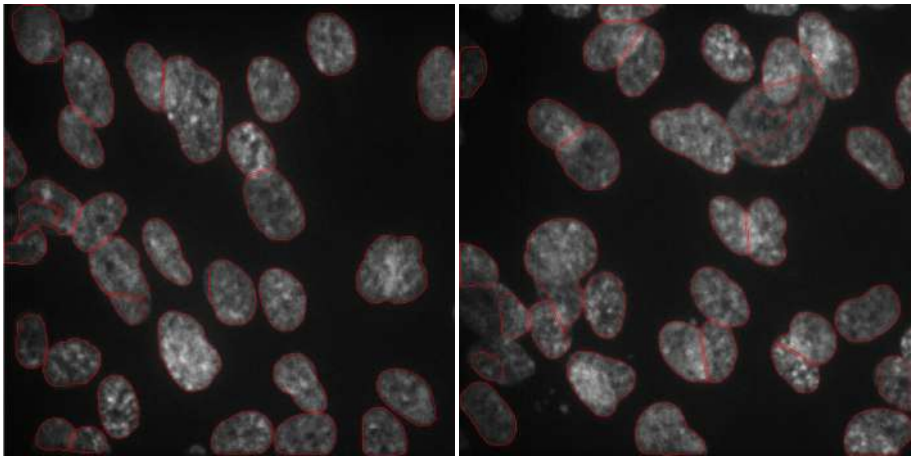

B

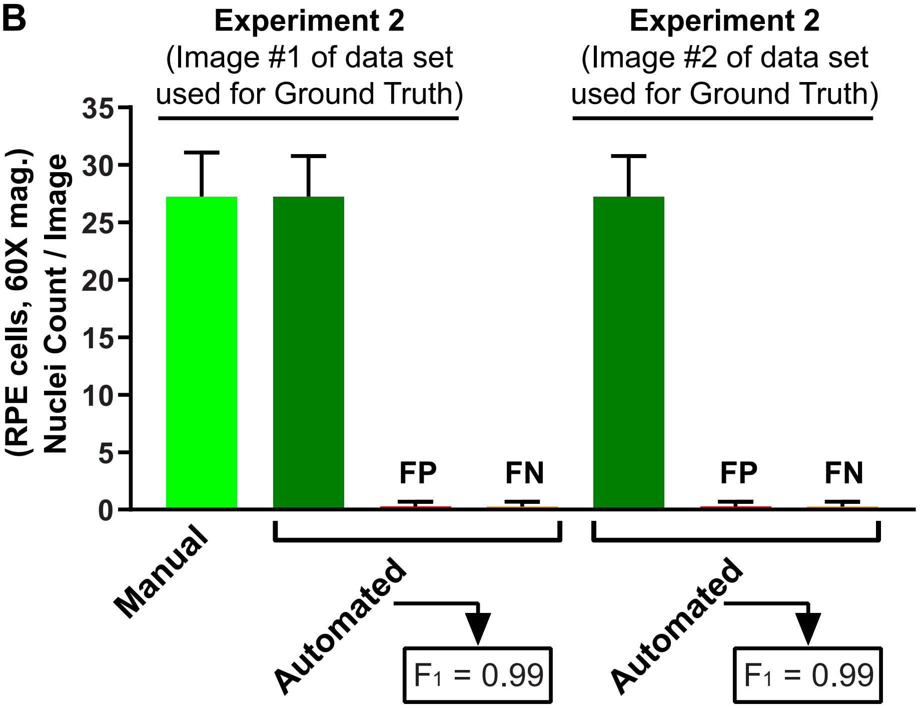

C

**RPE cells (Nuclei Count)**	**Experiment 2** (Ground Truth #1)	**Experiment 2** (Ground Truth #2)
Images Analyzed	8	8
Manual	27.3±3.8	27.3±3.8
Automated	27.3±3.5 **(100%)**	27.3±3.5 **(100%)**
FP	0.3±0.3 **(0.9%)**	0.3±0.4 **(0.9%)**
FN	0.3±0.4 **(0.9%)**	0.3±0.4 **(0.9%)**
Precision (α)	0.99	0.99
Recall (β)	0.99	0.99
**F1 score**	**0.99**	**0.99**
Accuracy	++++	++++

D

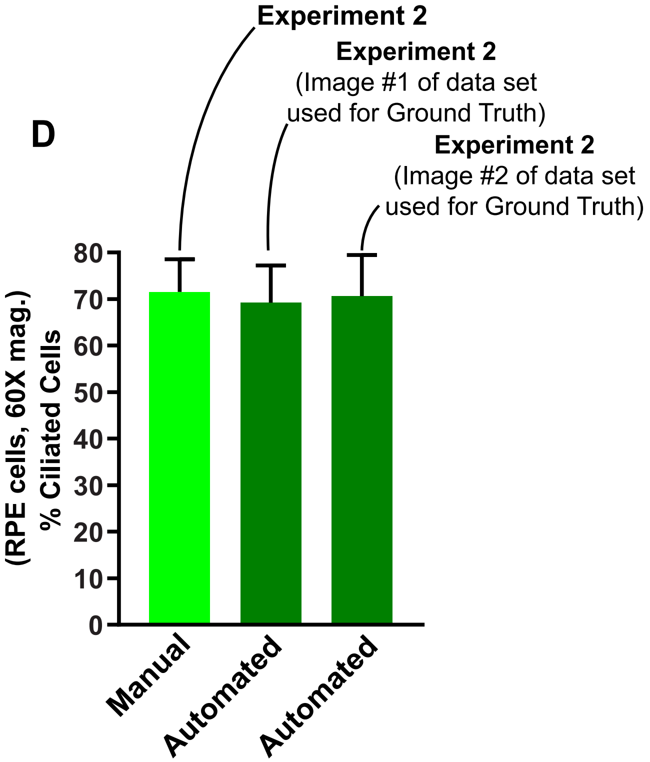

Supplement: Supplementary file 4 — Additional file 4: Figure S4. Quantitative analysis of ACDC accuracy in detecting nuclei and cilia frequency. (A) Examples of nuclei detection with ACDC software of the two representative images used for Experiment 2. (B) Manual and automated detection of RPE nuclei labeled with DAPI in 60×-mag. images. Manually-detected nuclei represent the total number of true nuclei (light green bar). Automatedly-detected nuclei represent the proportion of true nuclei that were detected in automated analysis mode (dark green bar). In regards to nuclei detection, repeating the analysis using two different representative images from the same Experiment 2 data set yielded identical FP and FN rates (0.9%) and identical F1 scores (F1 = 0.99). Note that detection auto-optimization from representative images is not utilized for nuclei segmentation and detection. (C) Tabular data for the values depicted in (B). Accuracy ratings are based on F1 score values, which range from 0 to 1.00 (0.95–1.00, ++++ ; 0.90–0.94, +++; 0.85–0.89, ++/+++). (D) Manual and automated analysis of cilia frequency for the images from Experiment 2. Cilia frequency values were calculated by dividing the cilia count/image values by the nuclei count/image values. All measurements are reported as averages ± standard deviations of multiple images from one experiment. [file 13630_2019_61_MOESM4_ESM.pdf]
