# Supplementary material for: New software for automated cilia detection in cells (ACDC)
Source: Cilia. 2019 Aug 1;8:1. doi: 10.1186/s13630-019-0061-z (PMC6670212; doi:10.1186/s13630-019-0061-z)

A

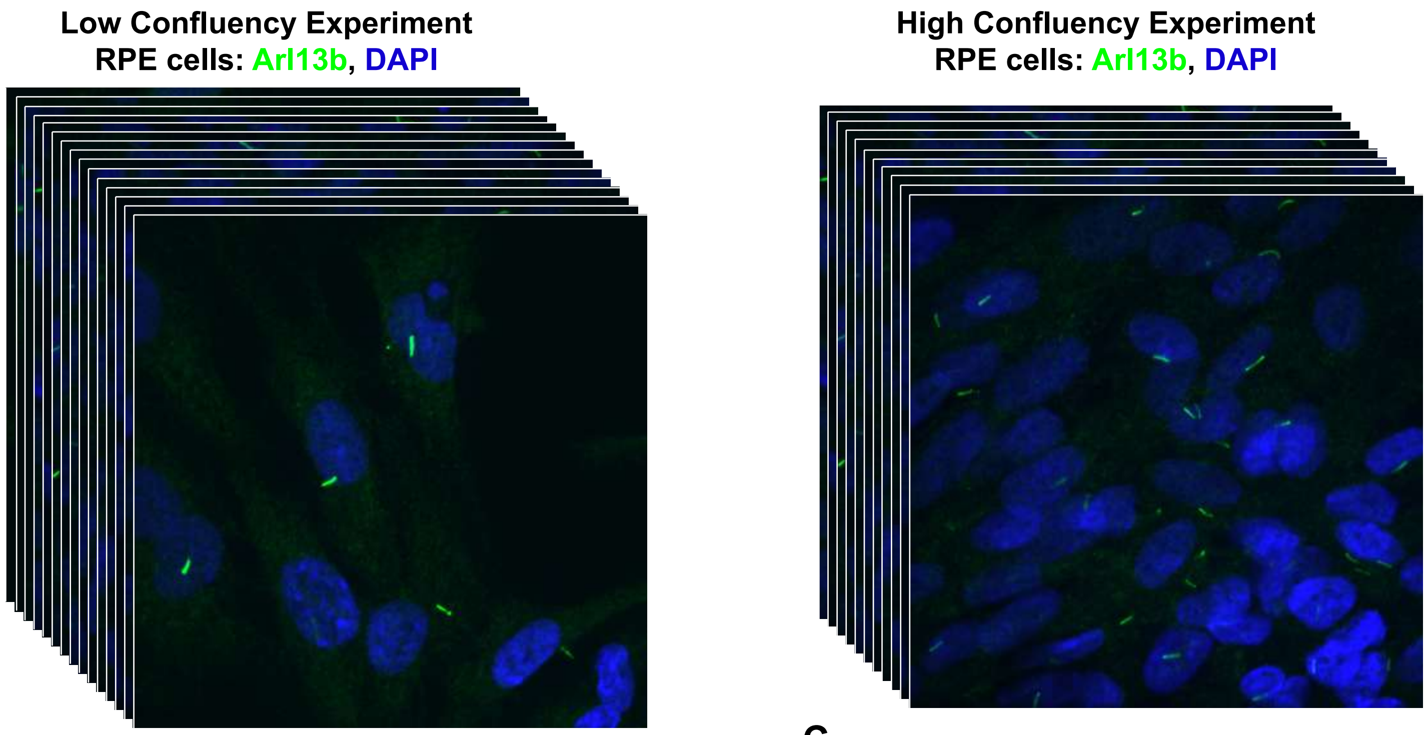

B

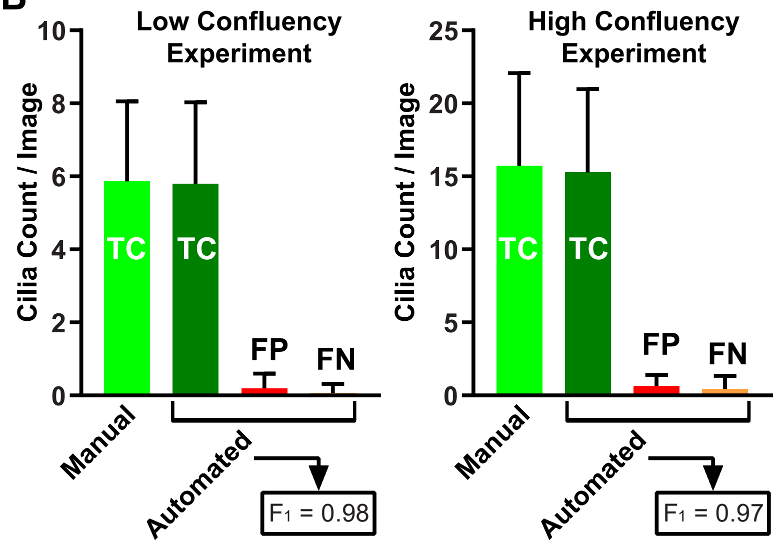

C

RPE cells (Cilia Count)	Low Confluency Experiment	High Confluency Experiment
Images Analyzed	15	11
Manual TC	5.9±2.2	15.7±6.4
Automated TC	5.8±2.2 **(99%)**	15.3±5.7 **(97%)**
FP	0.2±0.4 **(3%)**	0.6±0.8 **(4%)**
FN	0.1±0.2 **(1%)**	0.5±0.9 **(3%)**
Precision (α)	0.97	0.96
Recall (β)	0.99	0.97
**F1 score**	**0.98**	**0.97**
Accuracy	++++	++++

D

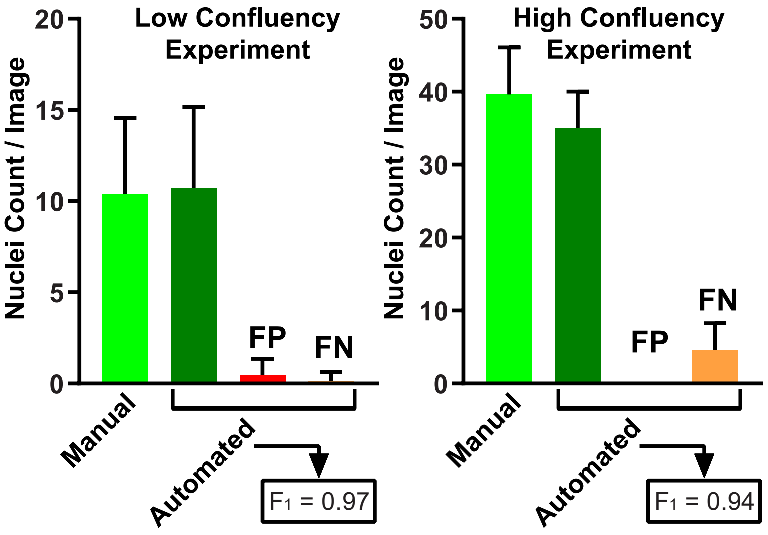

E

RPE cells (Nuclei Count)	Low Confluency Experiment	High Confluency Experiment
Images Analyzed	15	11
Manual	10.4±4.2	39.6±6.4
Automated	10.7±4.4 **(103%)**	35.0±5.0 **(88%)**
FP	0.5±0.9 **(4.5%)**	0.0±0.0 **(0%)**
FN	0.1±0.5 **(1%)**	4.6±3.6 **(12%)**
Precision (α)	0.96	1.00
Recall (β)	0.99	0.88
**F1 score**	**0.97**	**0.94**
Accuracy	++++	+++

F

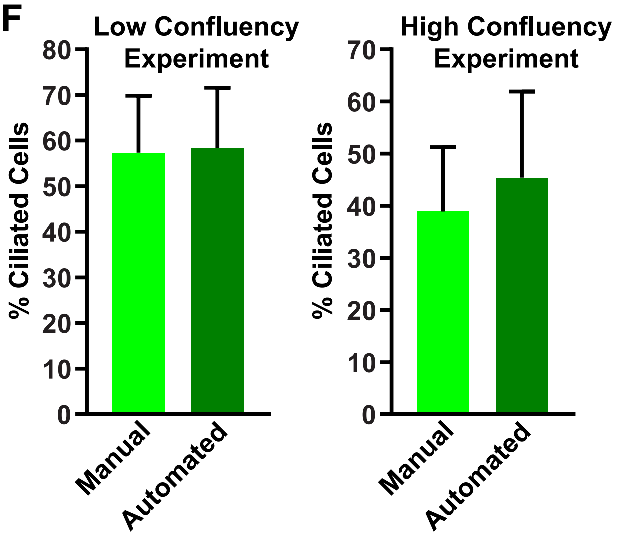

Supplement: Supplementary file 5 — Additional file 5: Figure S5. High cell confluency can affect automated cilia and nuclei detection and cell frequency measurements. (A) Analyzed images from two separate samples of differing cell confluency (low and high). (B and C) Bar graphs and corresponding tabular data of manual and automated analysis of RPE Arl13b-labeled cilia in 60×-mag. images. Manual true candidates (TC) represent the total number of true cilia (light green bar). Automated TC represents the number of manual TC that was detected in automated analysis mode (dark green bar). Despite differences in cell confluency, FP and FN rates for cilia detection remained between 1 and 4% and F1 scores were similar (low confluency, F1 = 0.98 vs. high confluency, F1 = 0.97). (D and E) Bar graphs and corresponding tabular data of manual and automated analysis of RPE nuclei in the same 60×-mag. images. FP rates for nuclei detection were greater in low confluency images than in high confluency images (4.5% vs. 0%), but FN rates for nuclei detection were much greater in high confluency images than in low confluency images (12% vs. 1%). Consequently, the large FN rate of 12% for nuclei detection in high confluency images yielded a worse accuracy score (F1 = 0.94) compared to that of low confluency images (F1 = 0.97). (F) Manual and automated analysis of cilia frequency for the low and high confluency data sets. Cilia frequency was calculated by dividing the cilia count per image values by the nuclei count per image values. Cilia frequency remained nearly identical between manual and automated analysis for low confluency images, but cilia frequency values varied more between manual and automated analysis for high confluency images (manual, 39% ± 12% vs. automated, 45% ± 17%). This difference is due to the disparity in nuclei detection between manual and automated analysis for high confluency images. All measurements are reported as averages ± standard deviations of multiple images from one experiment. [file 13630_2019_61_MOESM5_ESM.pdf]
