# Supplementary material for: New software for automated cilia detection in cells (ACDC)
Source: Cilia. 2019 Aug 1;8:1. doi: 10.1186/s13630-019-0061-z (PMC6670212; doi:10.1186/s13630-019-0061-z)
Supplement: Supplementary file 6 — Additional file 6: Table S6. Tabular data of Fig. 7. Images of RPE cells with Arl13b-stained cilia, taken at two different magnifications, were analyzed manually, automatedly, and semi-automaticallywith the ACDC software. Analysis times were recorded for each image and then averaged. Analysis times for fully automated analysis (“Auto) are greatly faster than those of manual analysis. Standard deviations of fully automated analysis times are much smaller than those of semi-automated analysis times (“Semi-Auto”), which are smaller than those of manual analysis times. Analysis times include the time required to count nuclei, count cilia, and measure cilia length. All measurements are reported as averages ± standard deviations of multiple images from one experiment. [file 13630_2019_61_MOESM6_ESM.pdf]

(see Fig. 7)

Lauring et al. | Table S6

**RPE cells**	60X magnification images			40X magnification images		
**Arl13b-cilia**	**Manual**	**Auto**	**Semi-Auto**	**Manual**	**Auto**	**Semi-Auto**
Images Analyzed	25	25	25	5	193	193
Analysis Time / Image	145.3±23.1	5.2±0.1	15.1±8.0	476.0±30.0	5.6±0.3	24.2±14.0
Avg. Cilia Count / Image	10.9±6.5	10.9±6.5	10.9±6.5	27.5±12.0	27.5±16.4	27.5±16.4
